# Thermodynamic Insights on the Structure‐Property Relationships in Substituted Benzenes: Are the Pairwise Interactions in Tri‐Substituted Methyl‐Nitro‐Benzoic Acids Still Valid?

**DOI:** 10.1002/cplu.202400703

**Published:** 2025-02-11

**Authors:** José M. Silva Ferraz, Vladimir N. Emel'yanenko, Dzmitry H. Zaitsau, Artemiy A. Samarov, Bruno Brunetti, Andrea Ciccioli, Stefano Vecchio Ciprioti, Sergey P. Verevkin

**Affiliations:** ^1^ Department of Basic and Applied Sciences for Engineering (S.B.A.I.) Sapienza University of Rome Via del Castro Laurenziano 7, Building RM017 00161 Rome Italy; ^2^ Competence Centre CALOR, Department Life, Light & Matter Faculty of Interdisciplinary Research University of Rostock 18059 Rostock Germany; ^3^ Institute of Technical Thermodynamics University of Rostock 18059 Rostock Germany; ^4^ FVTR GmbH 18059 Rostock Germany; ^5^ Saint Petersburg State University Peterhof 198504 Saint Petersburg Russian Federation; ^6^ Istituto per lo Studio dei Materiali Nanostrutturati, Consiglio Nazionale delle Ricerche Department of Chemistry Sapienza University of Rome P.le Aldo Moro 5 00185 Rome Italy; ^7^ Department of Chemistry Sapienza University of Rome P.le Aldo Moro 5 00185 Rome Italy

**Keywords:** benzoic acid derivatives, thermochemistry, phase transitions, quantum chemistry, structure-property relationships

## Abstract

A comprehensive experimental thermochemical study of nine methyl‐substituted nitrobenzoic acids was carried out, leading to the final standard molar enthalpies of formation in the gas phase. The combustion energies were measured using high‐precision combustion calorimetry, and the enthalpies of formation of the crystal phase were derived. The sublimation enthalpies were obtained from the vapor pressure‐temperature dependencies measured using the classic Knudsen effusion mass loss and the transpiration methods. The standard molar enthalpies of vaporisation were derived from the temperature dependence of the mass‐loss rates measured using the non‐isothermal thermogravimetry. The thermal behaviour, including melting temperatures and standard molar enthalpies of fusion, was investigated by DSC. The high‐level quantum chemical G* methods were used for the mutual validation of the experimental and theoretical gas phase enthalpies of formation of methyl‐substituted nitrobenzoic acids. The consistent set of experimental properties at the reference temperature *T*=298 K was evaluated and recommended for thermochemical calculations. The pairwise interactions of the substituents on the benzene ring were derived from nitro‐toluenes, methyl‐benzoic acids and nitro‐benzoic acids available in the literature, and the additivity of the contributions when three substituents are placed simultaneously in the benzene ring was discussed.

## Graphical abstract

A comprehensive thermodynamic study of nine methyl‐nitro‐benzoic acids was carried out. By combining experimental, empirical and computational methods, a consistent and reliable set of phase transition enthalpies and enthalpies of formation was established. These results were used to extend the “centerpiece” approach to estimate the gas‐phase enthalpies formation and to derive and analyse the pairwise interactions of the substituents on the benzene ring. It was found that only the sum of the pairwise interactions is sufficient to predict the gas phase formation enthalpies of the trisubstituted benzenes (with methyl, nitro and carboxyl substituents).

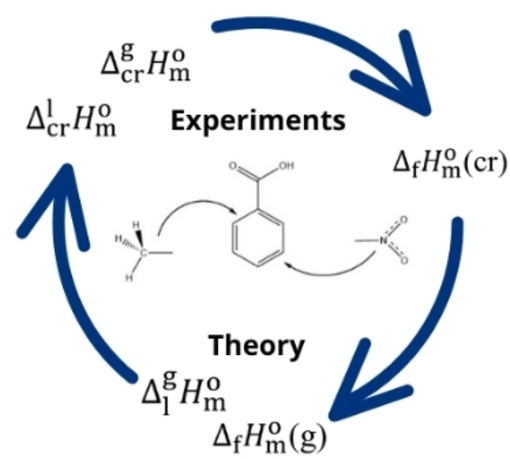

## Introduction

Thermodynamics is crucial to understanding the nature of substituted benzenes, as it helps predict how substituents on the benzene ring affect stability, reactivity, and interaction with other molecules.^[1]^ Thermodynamics generally determine whether reactions involving substituted benzenes, such as nitration, sulfonation, or halogenation, are thermodynamically favourable.^[2]^ For example, electron‐donating groups make the benzene ring more reactive towards electrophilic aromatic substitution, while electron‐withdrawing groups make it less reactive. Thermodynamic parameters help to quantify this reactivity.^[3]^ Thermodynamic properties influence the equilibria of substituted benzenes in different solvents, which is relevant for syntheses and industrial processes.^[2]^ Thermodynamics helps to understand the environmental behaviour of substituted benzenes, which can be pollutants.^[4]^ It also aids in predicting thermodynamic stability and degradation pathways.^[5]^ Chemists can thus assess how these compounds persist in the environment and develop methods to mitigate their effects.

**Figure 1:**
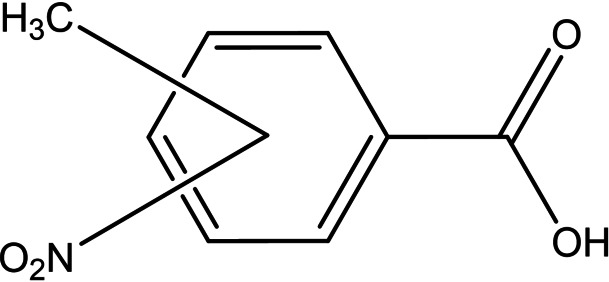
General structure of methyl‐substituted nitrobenzoic acid studied in this work: 3‐methyl‐2‐nitrobenzoic acid (3M2NBA), 4‐methyl‐2‐nitrobenzoic acid (4M2NBA), 5‐methyl‐2‐nitrobenzoic acid (5M2NBA), 6‐methyl‐2‐nitrobenzoic acid (6M2NBA), 2‐methyl‐3‐nitrobenzoic acid (2M3NBA), 4‐methyl‐3‐nitrobenzoic acid (4M3NBA), 2‐methyl‐5‐nitrobenzoic acid (2M5NBA), 2‐methyl‐4‐nitrobenzoic acid (2M4NBA), and 3‐methyl‐4‐nitrobenzoic acid (3M4NBA).

The investigation of the thermodynamic properties of substituted benzenes is one of the long‐standing goals of our laboratories. For example, the thermodynamic properties of dinitrobenzoic acids,^[5]^ dihydroxybenzoic acids^[6]^ and dibromobenzoic acids^[4]^ were investigated at Sapienza University of Rome.^[3]^ The thermochemical properties of methylbenzoic acids^[7]^ and nitrotoluenes^[8]^ were measured at the University of Rostock. This work reports a comprehensive study of the nine isomers of methyl nitrobenzoic acids (Figure 1).

This study includes the results of vapour pressure measurements, thermogravimetry, combustion calorimetry and differential scanning calorimetry. Only a few experimental studies on methyl‐nitrobenzoic acids have been reported. Initially by Monte et al.^[9]^ and later by Shelley et al..^[10]^ The available results were collected and validated along with our new results using independent experimental methods and empirical structure‐property correlations. The thermochemical property targeted in this work is the gas‐phase enthalpy of formation, $\Delta_{f}H_{m}^{o}$ (g), which is obtained by combining the results of combustion calorimetry and the results of vapour pressure measurements. The high‐level quantum chemical calculations were carried out to validate the experimentally obtained gas‐phase enthalpies of formation.

The experimental $\Delta_{f}H_{m}^{o}$ (g) values of the nine methylnitrobenzoic acids allowed us to expand our knowledge of the effects of substituents on the benzene ring. Indeed, the reliable thermochemical properties of the disubstituted benzenes (methylbenzoic acids,^[7]^ nitrotoluenes,^[8]^ and nitrobenzoic acids[^11^, ^12^]) determine the energetics of the pairwise interactions of the substituents on the benzene ring. What about trisubstituted benzenes? Are the pair‐wise interactions still valid for predicting the energetics of molecules using the rules of additivity, or do new additional effects arise from the agglomeration of the substituents on the benzene ring? This knowledge is essential for a correct prediction of the thermodynamic properties of multiply substituted benzenes, since the thermodynamic data can indicate whether the synergy of the interactions of the substituents stabilises (e. g. through resonance or inductive effects) or destabilises the benzene ring, making it more reactive. Thus, this understanding provides crucial insights into the stability, reactivity and interactions of substituted benzenes.

In this paper, we have used the general thermodynamic workflow (further details in ESI), which consists of the following five steps.

– **Step I**: the absolute vapour pressures were measured using the Knudsen effusion and transpiration methods. They were further combined with the available literature data in order to evaluate the consistent and reliable data for sublimation thermodynamics of the methyl‐nitrobenzoic acids.
– **Step II**: the thermal behaviour of the methyl‐nitrobenzoic acids was systematically studied using differential scanning calorimetry (DSC) and the experimental enthalpies of fusion were used to reconcile the sublimation and vaporisation thermodynamics.
– **Step III**: the standard molar enthalpies of formation in the crystalline phase, $\Delta_{f}H_{m}^{o}$ (cr), were derived using combustion calorimetry and combined with the sublimation enthalpies from Step I to create a consistent and reliable set of experimental gas‐phase enthalpies of formation.
– **Step IV**: the high‐level QC methods were used to calculate the theoretical gas‐phase enthalpies of formation, $\Delta_{f}H_{m}^{o}$ (g), to mutually validate the experiment and theory.
– **Step V**: the validated $\Delta_{f}H_{m}^{o}$ (g, 298 K) values for ten methyl nitrobenzoic acid isomers were used to analyse the energetics of substituent accumulation on the benzene ring and to explore the limits of group additivity for the mono‐, di‐, and tri‐substituted benzenes.

## Results and Discussion

### Step I: Vapour Pressure Temperature Dependencies and Experimental Standard Molar Enthalpies of Sublimation

The vapour pressures, $p_{i}$ , of MNBAs measured at different temperatures *T* were approximated by the following equation:^[13]^

(1) $$R\cdot \;\ln\left(p_{i}{/p}_{ref}\right)=a+\;\frac{b}{T}+\Delta_{cr}^{g}C_{p,m}^{o}\cdot \;ln\left(\frac{T}{T_{0}}\right)$$

where *a* and *b* are adjustable parameters, the arbitrary temperature *T*_0_=298 K, *R*=8.314462 J^−1^ K^−1^ mol^−1^ is the molar gas constant, the reference pressure $p_{ref}=1\;\;Pa,\;$ and $\Delta_{cr}^{g}C_{p,m}^{o}$ = $C_{p,m}^{o}$ (g) – $C_{p,m}^{o}$ (cr) is the difference between the standard molar heat capacities of the gaseous $C_{p,m}^{o}$ (g) and the crystalline phase $C_{p,m}^{o}$ (cr), respectively. The experimental absolute vapour pressures and the approximation parameters of Eq. (1) are given in Tables S1 (Knudsen method) and Table S2 (transpiration method).

**Figure 2:**
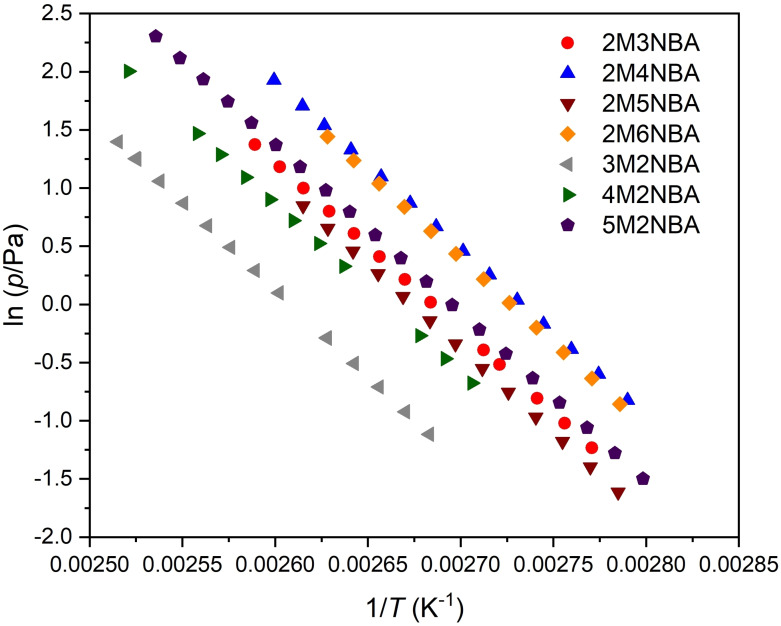
Plot of ln(*p)* vs 1/*T* obtained by the Knudsen effusion mass loss method for methyl nitrobenzoic acids using the cell with a 0.2 mm orifice.

The plots of ln(*p*/Pa) against 1/*T* were obtained by the Knudsen effusion mass loss method (KEML) for methyl nitrobenzoic acids using the cell with a 0.2 mm orifice are shown in Figure 2.

An initial classical KEML sublimation study of six methyl‐nitrobenzoic acids, was reported by Monte and Hillesheim.^[9]^ We used their reliable results to optimise the experimental procedure for the Knudsen measurements in Rome. Our new results are generally in agreement with the results of Monte and Hillesheim,^[9]^ as can be seen in Table 1. The complementary vapour pressure measurements of all nine methyl‐nitrobenzoic acids were carried out in this work in Rostock using the transpiration method. In this context, perspiration is one of the most suitable methods for studying thermally labile compounds, since mass transport during the experiment is carried out with nitrogen, which, in fact, is an inert gas, so the sample in the saturator is always protected. The investigation of methyl‐substituted nitrobenzoic acids, for which reliable data are already available in literature, not only made it possible to increase the list of MNBAs from six to nine samples but also contributed to the modification and refinement of the experimental transpiration procedure.

**Table 1:** Compilation of the experimental enthalpies of sublimation, $\Delta_{cr}^{g}H_{m}^{o}$ , of methyl‐substituted nitrobenzoic acids.

Compound	M^a^	*T*‐ range	$\Delta_{cr}^{g}H_{m}^{o}$ (*T*_av_)	$\Delta_{cr}^{g}H_{m}^{o}$ (298 K)^b^	Ref.
		K	kJ ⋅ mol^−1^	kJ ⋅ mol^−1^	
3‐methyl‐2‐nitrobenzoic acid	K	371.2–385.1	121.7±1.1	124.5±2.7	^[9]^
	K‐0.2	372.7–397.6	124.4±1.8	127.4±3.6	Table S1
	T	390.0–446.6	121.7±0.8	125.7±1.1	Table S2
				**125.7±1.0** ^c^	average
4‐methyl‐2‐nitrobenzoic acid	K‐0.2	369.5–396.7	120.3±1.8	123.1±3.6	Table S1
	T	377.2–428.0	117.4±1.4	120.9±1.6	Table S2
				**121.3±1.5** ^c^	average
5‐methyl‐2‐nitrobenzoic acid	K	355.2–371.1	116.5±0.6	118.7±2.2	^[9]^
	K‐0.2	357.4–394.4	120.3±1.4	122.9±2.8	Table S1
	T	375.7–402.1	120.2±1.5	123.3±1.8	Table S2
				**121.7±1.3** ^c^	average
6−methyl−2−nitrobenzoic acid	K	355.2–369.2	117.8±1.2	120.0±2.2	^[9]^
(2−methyl−6−nitrobenzoic acid)	K‐0.2	358.9–380.5	121.3±2.8	123.7±5.6	Table S1
*cr II*	T	373.3−402.4	116.9±1.1	119.9±1.5	Table S2
				**120.1±1.2** ^c^	average
6‐methyl‐2‐nitrobenzoic acid (*cr I*)	T	408.4–427.3	112.9±1.3	116.9±2.1	Table S2
2‐methyl‐3‐nitrobenzoic acid	K	357.2–371.2	117.3±1.2	119.5±2.3	^[9]^
	K‐0.2	360.9–386.2	119.3±2.4	121.8±4.8	Table S1
	K‐1	329.1–362.8	122.1±1.4	123.7±2.8	Table S1
	K‐3	309.6–340.8	123.2±2.0	124.1±4.0	Table S1
	T	384.0–448.3	116.6±1.2	120.6±1.4	Table S2
				**121.1±1.0** ^c^	average
4‐methyl‐3‐nitrobenzoic acid	K	363.1–373.1	116.2±1.3	118.6±2.5	^[9]^
	KEMS			(74.7)	^[10]^
	T	389.4–438.8	117.3±1.4	121.2±1.6	Table S2
				**121.1±1.0** ^c^	average
6‐methyl‐3‐nitrobenzoic acid	K‐0.2	359.1–382.4	120.5±1.8	122.9±3.6	Table S1
(2‐methyl‐5‐nitrobenzoic acid)	K‐1	331.1–358.5	124.1±1.6	125.7±3.2	Table S1
	K‐3	315.7–330.4	120.1±3.2	120.9±6.4	Table S1
	T	403.7–438.9	114.3±1.2	118.4±1.6	Table S2
				**120.3±1.3** ^c^	average
2‐methyl‐4‐nitrobenzoic acid	K‐0.2	358.4–384.2	120.0±2.3	122.5±4.6	Table S1
	T	376.4–416.8	109.2±1.3	112.5±1.5	Table S2
				**113.5±1.4** ^c^	average
3‐methyl‐4‐nitrobenzoic acid	K	363.2–379.2	116.8±1.2	119.3±2.5	^[9]^
	KEMS			(66.0)	^[10]^
	T	398.6–437.7	121.0±0.9	125.0±1.3	Table S2
				**122.2±0.9** ^c^	average

^a^ Methods: K = Knudsen Effusion Mass Loss (orifice diameter of 0.2 mm = K‐0.2; orifice diameter of 1 mm = K‐1; orifice diameter of 3 mm = K‐3); T = transpiration method; KEMS = Knudsen Effusion Mass Spectrometry. ^b^ Vapour pressures available in the literature were treated using Eqs. 1–3 to calculate the enthalpies of sublimation at 298 K. The uncertainties of the sublimation enthalpies *U*($\Delta_{cr}^{g}H_{m}^{o}$ ) have been expanded to a 0.95 level of confidence. They include uncertainties from the fitting equation and uncertainties from temperature adjustment to *T*=298 K. ^c^ Weighted mean value (uncertainties were taken as the weighting factor). Recommended values are given in bold.

The standard molar enthalpies of sublimation of MNBAs at the average temperatures, *T*, were derived from the temperature dependence of vapour pressures approximated by Eq. (1) using the following equation:

(2) $$\Delta_{cr}^{g}H_{m}^{o}\left(T\right)=-\;b+\Delta_{cr}^{g}C_{p,m}^{o}\times T$$

where *b* is one of the adjustable parameters of Eq. (1). The standard molar sublimation entropies at temperatures *T* were also derived from the temperature dependences of vapour pressures (approximated by Eq. 1) as follows:

(3) $$\Delta_{cr}^{g}S_{m}^{o}\left(T\right)=\Delta_{cr}^{g}H_{m}^{o}/T+R\times ln\left(p_{i}/p^{o}\right)$$

$\Delta_{cr}^{g}H_{m}^{o}$ (298 K) at the reference temperature *T*=298 K of the MNBAs were calculated using Eqs. (1) to (3) with the $\Delta_{\mathrm{cr}}^{g}C_{p,m}^{o}$ =−33.9 J ⋅ mol^−1^ ⋅ K^−1^ (derived from the $C_{p,m}^{o}$ (cr)=220.8 J ⋅ mol^−1^ ⋅ K^−1^, estimated using empirical method by Chickos *et al*.^[14]^). The uniformly treated results for MNBAs are summarized in Table 1 for comparison.

Our new results for the sublimation enthalpies, measured using KEML and transpiration methods, agree well with each other and with the results of Monte and Hillesheim^[9]^ (Table 1). To gain more confidence in the experimental sublimation enthalpies, the weighted mean (uncertainties were used as weighting factors) was calculated for each isomer and recommended for thermochemical calculations. It should also be mentioned, however, that the sublimation enthalpies of 4‐methyl‐3‐nitrobenzoic acid and 3‐methyl‐4‐nitrobenzoic acid are reported by Shelley *et al.*.^[10]^ (see Table 1), but the experimental details are not given in the original work and the results for both compounds are implausible.

### Step II: Diagnostics of Consistency of Phase Transitions

*Solid‐liquid and solid‐solid phase transitions*. The studied samples are solid at room temperature. The fusion enthalpies measured in this work and the fusion enthalpies taken from the literature are compared in Table 2. Indeed, the MNBAs exhibit limited thermal stability, which is true for all aromatic carboxylic acids. Nevertheless, sufficient agreement was found between the available results for each compound (except for 4‐methyl‐3‐nitrobenzoic acid, for which the result of Shelley *et al.*.^[10]^ is significantly lower for unclear reasons). Since the isomeric methylnitrobenzoic acids are generally similar in structure, a certain degree of similarity in their melting behaviour is to be expected. This similarity can be demonstrated using Walden′s rule for thermochemistry^[2]^ as follows. Paul Walden found^[16]^ that the ratio between the fusion enthalpy and the fusion temperature is remarkably constant for differently structured organic molecules:
(4)

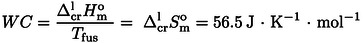




**Table 2:** Phase transition thermodynamics of methyl‐substituted nitrobenzoic acids (in kJ⋅mol^−1^)^a^.

Compounds	*Source* ^b^	*T*_fus_, K	$\Delta_{cr}^{l}H_{m}^{o}$ ^b^ at *T*_fus_	$\Delta_{cr}^{l}H_{m}^{o}$ ^*c*^	$\Delta_{cr}^{g}H_{m}^{o}$ ^d^	$\Delta_{l}^{g}H_{m}^{o}$ ^e^
				298 K		
3‐methyl‐2‐nitrobenzoic acid	^[9]^	494.9±0.2	37.8±0.5			
	A	498.4±0.8	35.4±1.0			
	B	497.3±0.2	34.9±0.5			
	B	497.8±0.2	34.4±0.5			
		**496.7±0.1** ^f^	**35.7±0.3** ^f^	**24.9±3.3**	**125.7±1.0**	**100.8±3.4**
						
4‐methyl‐2‐nitrobenzoic acid	A	428.4±0.3	24.4±0.3			
	B	426.8±0.5	25.6±0.5			
		**428.0±0.3** ^f^	**24.7±0.3** ^f^	**17.6±2.1**	**121.3±1.5**	**103.7±2.6**
						
5‐methyl‐2‐nitrobenzoic acid	^[9]^	408.5±0.2	22.6±0.5			
	A	409.8±0.3	21.5±0.4			
	A	408.8±0.3	20.2±0.5			
		**408.9±0.2** ^f^	**21.4±0.3** ^f^	**15.3±1.8**	**121.7±1.3**	**106.4±2.2**
						
6‐methyl‐2‐nitrobenzoic acid	A	403.9±0.8	1.8±1.0			
*cr II to cr I* ^*g*^	B	404.8±1.0	3.1±0.5			
	B	405.1±1.0	3.8±1.0			
		**404.5±0.5** ^f^	**3.0±0.4** ^f^			
						
*cr I to liq*	^[9]^	428.1±0.2	(27.0±0.5)			
	A	428.3±0.5	23.7±0.5			
	A	428.4±0.5	24.8±0.5			
	B	428.1±0.3	21.2±1.0			
	B	428.5±0.5	21.3±1.0			
		**428.3±0.2** ^f^	**23.7±0.3** ^f^			
		**428.3±0.2**	**26.7±0.5** ^h^	**19.6±2.2**	**120.1±1.2**	**100.5±2.5**
						
2‐methyl‐3‐nitrobenzoic acid	^[9]^	456.6±0.2	32.6±0.2			
	A	458.3±0.5	30.3±0.2			
		**456.8±0.2** ^f^	**31.5±0.2** ^f^	**23.0±2.6**	**121.1±1.0**	**98.1±2.8**
						
4‐methyl‐3‐nitrobenzoic acid	^[9]^	(459.8±0.2)	28.4±0.5			
	A	464.0±0.3	27.9±0.2			
		**464.2±0.3** ^f^	**28.0±0.2** ^f^	**18.9±2.7**	**120.4±1.4**	**101.5±3.0**
						
6‐methyl‐3‐nitrobenzoic acid	A	451.2±0.4	29.0±0.5			
						
(2‐methyl‐5‐nitrobenzoic acid)	B	451.4±0.2	26.9±0.3			
	B	451.5±0.2	26.9±0.3			
		**451.4±0.1** ^f^	**27.2±0.2** ^f^	**19.0±2.5**	**120.3±1.3**	**101.3±2.8**
						
2‐methyl‐4‐nitrobenzoic acid	A	428.4±0.2	21.4±0.5			
	A	428.9±0.2	21.0±0.5			
		**428.7±0.1** ^f^	**21.2±0.4** ^f^	**14.1±2.2**	**113.5±1.4**	**99.4±2.6**
						
3‐methyl‐4‐nitrobenzoic acid	^[9]^	489.1±0.2	33.8±0.5			
	^[10]^	492.4±0.5	35.4±1.0			
	A	491.1±0.3	33.5±0.5			
		**490.0±0.2** ^f^	**33.8±0.3** ^f^	**23.3±3.2**	**122.2±0.9**	**98.9±3.3**

^a^ Uncertainties are presented as expanded uncertainties (0.95 level of confidence with k=2). ^b^ The thermal behavior reported in the literature or studied in this work as follows: A = Rostock with Perkin‐Elmer Pyris Diamond DSC; B = Rostock with Mettler Toledo DSC 822e; The experimental enthalpies of fusion $\Delta_{cr}^{l}H_{m}^{o}$ measured at *T*_fus_ were adjusted to *T*=298 K with help of the equation: $\Delta_{\mathrm{cr}}^{l}H_{m}^{o}$ (298 K)/(J ⋅ mol^−1^)= $\Delta_{\mathrm{cr}}^{l}H_{m}^{o}$ (*T*_fus_/K)−($\Delta_{\mathrm{cr}}^{g}C_{p,m}^{o}$ −$\Delta_{l}^{g}C_{p,m}^{o}$ )×[(*T*_fus_/K)−298 K] where $\Delta_{\mathrm{cr}}^{g}C_{p,m}^{o}$ =−33.9 J ⋅ mol^−1^ ⋅ K^−1^ and $\Delta_{l}^{g}C_{p,m}^{o}$ =−88.5 J ⋅ mol^−1^ ⋅ K^−1^ were estimated from $C_{p,m}^{o}$ (cr)=220.8 J ⋅ mol^−1^ ⋅ K^−1^ and $C_{p,m}^{o}$ (liq)=299.7 J ⋅ mol^−1^ ⋅ K^−1^ according to the empirical procedures developed by Chickos et al.^[15]^. Uncertainties in the temperature adjustment of fusion enthalpies from *T*_fus_ to the reference temperature are estimated to account with 30 % to the total enthalpic adjustment.^[14]^ ^c^ The experimental enthalpies of fusion $\Delta_{cr}^{l}H_{m}^{o}$ at the reference temperature *T*=298 K. ^d^ Experimental values evaluated in Table 1. ^e^ Calculated as the difference of column 6 and 5 in this table. ^f^ Weighted average value (the uncertainty was taken as the weighing factor). Values in parenthesis were disregarded by averaging. Values highlighted in bold were used for thermochemical calculations. ^g^ Solid‐solid phase transition, $\Delta_{Phase\;II}^{Phase\;I}H_{m}^{o}$ =3.0 kJ⋅mol^−1^. For comparison $\Delta_{Phase\;II}^{Phase\;I}H_{m}^{o}$ =$\Delta_{cr}^{g}H_{m}^{o}$ (Phase II)−$\Delta_{cr}^{g}H_{m}^{o}$ (Phase I)=119.9−116.9=3.0±2.6 kJ⋅mol^−1^ from the transpiration data shown in Table 1. ^h^ Calculated as the sum of the solid‐solid and solid‐liquid phase transitions for 6‐methyl‐2‐nitrobenzoic acid given in this table.

This observation was based on experimental results of 35 compounds (mostly benzene derivatives like aniline, dimethylaniline, nitrobenzene, diphenylmethane, diphenylamine, acetophenone, *etc*., but also anhydrides, aliphatic esters, *etc*.). The value *WC*=56.5 J ⋅ K^−1^ ⋅ mol^−1^ is known as *W*alden's *C*onstant.^[17]^ According to Walden, the prerequisite for this constancy is that the compounds do not associate in the liquid state. In our most recent work, however, we have shown that even for the five nucleobases adenine, thymine, cytosine, guanine and uracil, which have relatively high melting temperatures and a high degree of association in the liquid phase, the *WC* values are very close to the “classical” Walden value.^[17]^ Thus, *WC* can be considered a useful tool to check the internal consistency of fusion enthalpies within a series of structurally similar molecules and is expected to be invariant within this series. Indeed, the ratios (fusion enthalpy/fusion temperature) calculated from the data in Table 2 for MNBAs show a remarkable constancy WC=61.5±2.6 J ⋅ K^−1^ ⋅ mol^−1^ (Table S3) This value is not far from the “classical” *WC*, but the most important conclusion is that the DSC and vapour pressure measurements were performed correctly and were not influenced by thermal degradation.

According to our DSC studies, a solid‐solid phase transition, $\Delta_{Phase\;II}^{Phase\;I}H_{m}^{o}$ =3.0±0.4 kJ⋅mol^−1^ at 404.5±0.5 K was found for 6‐methyl‐2‐nitrobenzoic acid. This phase transition was apparently overlooked by Monte *and* Hillesheim^[9]^ (see Table 2). In this work, we deliberately measured the vapour pressures for this compound above and below 404.5 K using the transpiration method. The difference between the enthalpy of sublimation of 6‐methyl‐2‐nitrobenzoic acid $\Delta_{cr}^{g}H_{m}^{o}$ (Phase II, 298 K)=119.9±1.5 kJ ⋅ mol^−1^ in the Phase II and $\Delta_{cr}^{g}H_{m}^{o}$ (Phase I, 298 K)=116.9±2.1 kJ ⋅ mol^−1^ in Phase I makes it possible to determine the enthalpy of the solid‐solid phase transition independently $\Delta_{Phase\;II}^{Phase\;I}H_{m}^{o}$ =$\Delta_{cr}^{g}H_{m}^{o}$ (Phase II)–$\Delta_{cr}^{g}H_{m}^{o}$ (Phase I)=119.9–116.9=3.0±2.6 kJ⋅mol^−1^ and this result is indistinguishable from the DSC result $\Delta_{Phase\;II}^{Phase\;I}H_{m}^{o}$ =3.0±0.4 kJ⋅mol^−1^ in Table 2. This empirical support for the results of solid‐liquid and solid‐solid phase transitions ensures the experimental values listed in Tables 1 and 2.

*Solid‐Gas and Liquid‐Gas Phase Transitions*. Before the sublimation enthalpies evaluated in Table 1 can be considered reliable and recommended for thermochemical calculations, these results should be validated by independent experimental and empirical methods.

The experimental standard molar enthalpies of vaporisation, $\Delta_{l}^{g}H_{m}^{o}$ (298 K), can serve as an independent check for the experimental standard molar enthalpies of sublimation, $\Delta_{cr}^{g}H_{m}^{o}$ (298 K). The $\Delta_{l}^{g}H_{m}^{o}$ (298 K)‐values, were derived in this section for all nine methyl‐nitrobenzoic acids as the difference between the standard molar enthalpies of sublimation, $\Delta_{cr}^{g}H_{m}^{o}$ (298 K), evaluated in Table 1 and the standard molar enthalpies of fusion, $\Delta_{cr}^{l}H_{m}^{o}$ (298 K), evaluated in Table 2:

(5) $$\Delta_{l}^{g}H_{m}^{o}\left(298\;K\right)=\Delta_{cr}^{g}H_{m}^{o}\left(298\;K\right)-\Delta_{cr}^{l}H_{m}^{o}\left(298\;K\right)$$

The resulting $\Delta_{l}^{g}H_{m}^{o}$ (298 K)‐values are given in Table 3, column 3, and were validated using the results measured with the non‐isothermal thermogravimetry (NITG) method (Table 3, column 2). The enthalpies of vaporisation measured by NITG are both reproducible and accurately measured at relatively high temperatures (Table S4), at which sufficient volatility can be achieved. However, the temperature adjustment of the measured values to the reference temperature contributes significantly to the uncertainties of the resulting values. As can be seen from Table 3, the $\Delta_{l}^{g}H_{m}^{o}$ (298 K)‐values of the NITG method agree with the values estimated according to Eq. (5) within the combined experimental uncertainties and independently confirm the results evaluated in Tables 1 and 2.

**Table 3:** Comparison of the enthalpies of vaporisation, $\Delta_{l}^{g}H_{m}^{o}$ , of methyl‐substituted nitrobenzoic acids determined with NITG, derived from the solid‐gas and solid‐liquid phase transitions and the values estimated using the “centerpiece” approach (at 298 K in kJ ⋅ mol^−1^)^a^ .

	$\Delta_{l}^{g}H_{m}^{o}$ ^b^	$\Delta_{l}^{g}H_{m}^{o}$ ^c^	$\Delta_{l}^{g}H_{m}^{o}$ ^d^	Δ ^e^
3‐methyl‐2‐nitrobenzoic acid	‐	100.8±3.4	104.4	*−3.6* ^f^
4‐methyl‐2‐nitrobenzoic acid	‐	103.7±2.6	103.0	0.7
5‐methyl‐2‐nitrobenzoic acid	103.4±2.7	106.4±2.2	105.6	0.8
6‐methyl‐2‐nitrobenzoic acid	100.4±2.9	100.5±2.5	101.1	*−0.6* ^f^
2‐methyl‐3‐nitrobenzoic acid	99.2±3.2	98.1±2.8	101.1	*−3.0* ^f^
4‐methyl‐3‐nitrobenzoic acid	‐	101.5±3.0	103.0	−1.5
6‐methyl‐3‐nitrobenzoic acid	99.8±3.2	101.3±2.8	101.1	0.2
2‐methyl‐4‐nitrobenzoic acid	‐	99.4±2.6	98.4	1.0
3‐methyl‐4‐nitrobenzoic acid	‐	98.9±3.3	101.7	−2.8

^a^ Uncertainties are presented as expanded uncertainties (0.95 level of confidence with k=2). ^b^ The experimental values from Table S4 measured using NITG. Uncertainties of the vaporisation enthalpies include uncertainties from the fitting equation and uncertainties from temperature adjustment to T=298 K. Uncertainties in the temperature adjustment of vaporisation enthalpies to the reference temperature *T*=298 K are estimated to account with 20 % to the total enthalpic adjustment. ^c^ The experimental values from Table 2 (column 7). ^d^ The values estimated using the “centerpiece” approach as shown in Figures S2 and S3. ^e^ Calculated as the difference of columns 3 and 4 in this table. ^f^ In the compounds shown in italics, the possibility of the “buttress” effects due to the 1,2,3‐sequence of substituents on the benzene ring, is expected.

The advantage of group‐additivity (GA) methods is that they are developed from validated experimental data. If the new results deviate significantly from the additive rules, they may be considered questionable or the reason for this unusual deviation should be adequately explained. If the new results follow the already established trends, they can be integrated into the network of existing reliable data. The empirical methods based on the principles of additivity are best suited for this purpose.^[18]^

Admittedly, the enthalpies of sublimation are not additive, but the enthalpies of vaporisation obey the rules of additivity.^[19]^ Therefore, the standard molar enthalpies of vaporisation, $\Delta_{l}^{g}H_{m}^{o}$ (298 K), derived according to Eq. (5) can be independently validated using the GA method, which leads to the desired validation of the experimental data evaluated in Tables 1 and 2.

**Figure 3:**
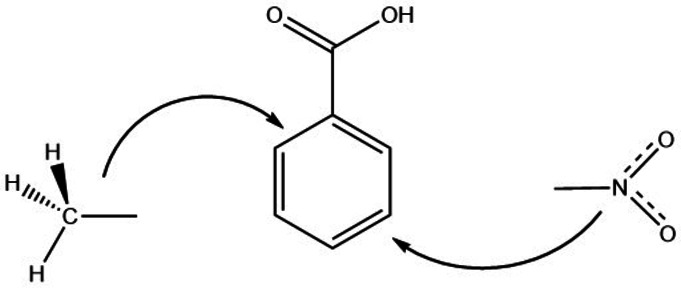
The general concept of the “centerpiece” approach.

The GA method essentially involves dividing the reliable experimental enthalpies (most commonly the enthalpy of vaporisation or the enthalpy of formation) of a possible large number of molecules into the smallest possible groups (increments) and developing the numerical values of the increments from the matrix of reliable data. The estimation is then obtained by adding the appropriate number and type of increments for the desired molecular structure. In fact, the addition of many increments is statistically prone to large uncertainties or even to systematic errors. To avoid this deficiency, the GA method can be improved by performing additive calculations from a structurally similar molecule (the so‐called “centerpiece”) and adding the essential increments or substituents stepwise to this “centerpiece”[^20^, ^21^] . In the context of this work, benzoic acid is the most suitable “centerpiece”. Methyl and nitro groups can then be attached to these “centerpiece” at different positions of the benzene rings (see Figure 3).

The thermodynamic properties of benzoic acid are well‐known (Table S5). The enthalpic contributions for the methyl substituent, Δ*H(*H*→ CH_3_*), and the nitro‐substituent, Δ*H(*H*→ NO_2_*), can be easily quantified (see Fig. S2) from the differences between the enthalpies of the methylbenzene (or nitrobenzene) and the enthalpies of the benzene itself. Using this scheme, the contributions for Δ*H(*H*→ CH_3_)* and Δ*H(*H*→ NO_2_),* were derived (Table 4) using thermodynamic data for benzene, methylbenzene, and nitrobenzene compiled in Table S5.

**Table 4:** Parameters and pairwise nearest and non‐nearest neighbour interactions of substituents on the benzoic acid as the “centerpiece” for calculation of thermodynamic properties of methyl‐nitrobenzoic acids at 298 K (in kJ⋅ mol^−1^).

Contribution	$\Delta_{l}^{g}H_{m}^{o}$ ^a^	$\Delta_{f}H_{m}^{o}$ (g) ^b^
benzene	33.9	82.9
toluene	38.1	50.1
nitrobenzene	55.0	65.6
benzoic acid	75.8	−294.1
ΔH(H→CH_3_)	4.2	−32.8
ΔH(H→NO_2_)	21.1	−17.3
		
ortho (CH_3_−NO_2_)	≈0	4.3
meta (CH_3_−NO_2_)	≈ 0	−3.8
para (CH_3_−NO_2_)	1.2	−2.9
		
ortho (CH_3_=COOH)	≈ 0	6.6
meta (CH_3_=COOH)	3.3	−2.0
para (CH_3_=COOH)	1.9	−1.6
		
ortho (NO_2=_COOH)	≈ 0	31.1
meta (NO_2_=COOH)	≈ 0	7.4
para (NO_2_=COOH)	−2.7	3.9

^a^ The values were calculated as shown in Figure S2 using the experimental data from Table S5. ^b^ The values were calculated as shown in Figure S3 using the experimental data from Table S5.

These enthalpic contributions Δ*H(*H *→ CH_3_)* and Δ*H(*H*→ NO_2_),* can be now applied to construct a general framework of the nitro‐benzoic acid and methyl‐nitro‐benzoic acid (Fig. S2). In most cases, this framework can already make a rough energy‐related prediction of the level of the vaporisation or formation enthalpies. However, this framework must be improved by adding the specific contributions for the mutual interactions between the carboxyl, methyl and nitro substituents present in the molecule in order to provide a more accurate prediction.

The mutual pairwise enthalpic interactions of the substituents in the benzene ring can be attributed to three types of contribution that are specific to the *ortho*‐, *para‐* and *meta*‐positions of the substituents placed in the benzene ring. Figure S2 shows how pairwise interactions can be derived. For example, to quantify the enthalpic contribution “*para (NO_2_ – COOH)*” for the non‐bonded interaction of the nitro‐group with the carboxyl substituent in the *para*‐position on the benzoic acid (considered as the “centerpiece”), we must first construct the “theoretical framework” of 4‐nitro‐benzoic acid. To do this, we simply add the contribution Δ*H(*H*→ NO_2_)* from Table 4 to the experimental enthalpy (enthalpy of vaporisation or enthalpy of formation) of benzoic acid from Table S5. This “theoretical framework” of 4‐nitro‐benzoic acid does not include the “*para (NO_2_ – COOH)*” interaction. However, this interaction is present in the real 4‐nitro‐benzoic acid (symbolised by an arrow in Figure S2). The arithmetic difference between the experimental enthalpy (enthalpy of vaporisation or enthalpy formation) of 4‐nitro‐benzoic acid and the enthalpy of the “theoretical framework” therefore directly provides the quantitative size of the pairwise interaction “*para (NO_2_ – COOH)*” directly (see Table 4). Using the same logic, the enthalpic contributions for “*ortho (NO_2_ – COOH)*” and “*meta (NO_2_ – COOH)*” shown in Figure S2 can be derived (see Table 4) with benzoic acid and the parameter Δ*H(*H*→ NO_2_)*. In the same way, the enthalpic contributions of pairwise *ortho‐ meta‐* and *para‐*interactions of methyl and carboxyl substituents, as well as of methyl‐ and nitro‐substituents, were derived and are summarized in Table 4.

As can be seen from this table, the contributions of pairwise interactions of substituents to the enthalpy of vaporisation were found to be negligible in many cases, however, in other instances, they also lie in the range from −2.7 to 3.3 kJ/mol. However, the discussion of the magnitudes of the pairwise interactions with respect to $\Delta_{l}^{g}H_{m}^{o}$ is rather limited, since these contributions reflect the density of the molecular packing in the liquid. These contributions are not negligible and must be considered as empirical constants for the correct prediction of the enthalpy of vaporisation.

**Figure 4:**
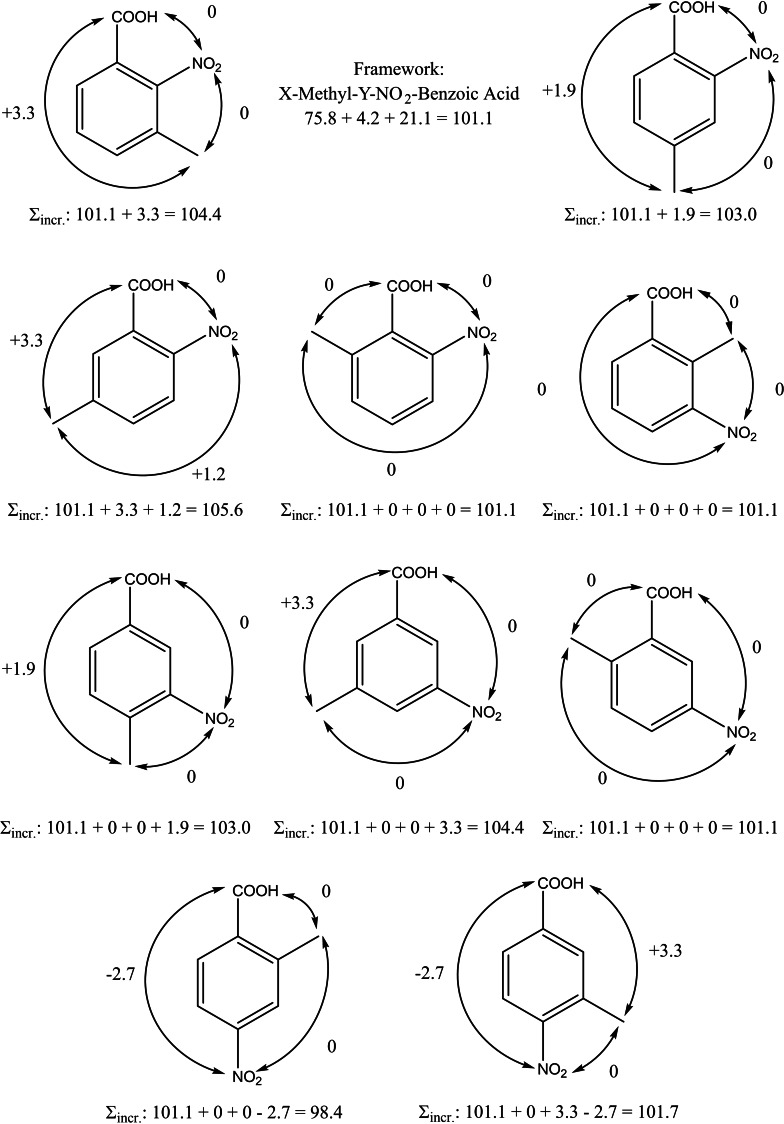
Calculation the enthalpies of vaporisation, $\Delta_{l}^{g}H_{m}^{o}$ (298 K), of MNBAs using benzoic acid as the “centerpiece”. All increments and pairwise interactions are from Table 4 and Figure S2 (in kJ⋅mol^−1^).

For the MNBAs, the “theoretical framework” required for the energetic calculations is generated by adding the contributions Δ*H(*H*→CH_3_)* and Δ*H(*H*→NO_2_)* to the enthalpy of the benzoic acid (see, for example, Figure 4 for the enthalpies of vaporisation). To predict the proposed properties of this trisubstituted benzene, the nearest (*ortho*‐) and non‐nearest (*meta*‐ and *para*‐) pairwise interactions should be loaded into this theoretical framework. For example, to estimate the enthalpy of vaporisation for 5‐methyl‐2‐nitro‐benzoic acid, the *ortho (NO_2_−COOH), meta (CH_3_−COOH)*, and *para (CH_3_−NO_2_*) contributions should be added (see Figure 4). In this way, the values of the additive enthalpies of vaporisation were estimated for all nine methyl‐nitrobenzoic acids (Table 3 entry 4) and compared with the experimental enthalpies of vaporisation obtained according to Eq. (5) (Table 3 entry 3). As can be observed, most experimental and additive values agree within ±1 kJ mol^−1^, which is even better than the uncertainty associated with the experimental values of ±(2 to 3) kJ mol^−1^. However, for two compounds, 3‐methyl‐2‐nitrobenzoic acid and 2‐methyl‐3‐nitrobenzoic acid, there was an overestimation of the vaporisation enthalpies by ≈3 kJ mol^−1^. It is important to note that, the aforementioned molecules (and 6‐methyl‐2‐nitrobenzoic acid) exhibit a structural peculiarity that can cause an additional “buttress”^[22]^ interaction, which arises from the spatial restriction of the bulky substituents in the benzene ring (1,2,3 sequence). This specific molecular shape can lead to a less dense packing of the molecules in the liquid phase, which results in a decrease in the enthalpy of vaporisation. This can be observed in the experimental data for 3‐methyl‐2‐nitrobenzoic acid and 2‐methyl‐3‐nitrobenzoic acid when compared to the additive values. However, it should be acknowledged that this explanation is severely affected by the experimental uncertainty, which is just as large as the ‘buttress’ effect observed for both compounds. Furthermore, there is no evidence for a specific contribution of the 1,2,3‐sequence for 6‐methyl‐2‐nitrobenzoic acid.

In conclusion, the reasonable agreement of the experimental and GA derived enthalpies of vaporisation for all nine methyl‐nitrobenzoic acids should be considered a good indicator of the consistency and reliability of the enthalpies of the solid‐gas and solid‐liquid phase transitions previously evaluated.

### Step III. Enthalpies of Formation from Combustion Calorimetry

The specific energies of combustion, $\Delta_{c}u^{o}$ (cr), of high‐purity samples of methyl‐nitro‐benzoic acids were measured using combustion calorimetry (further detailed in Tables S6 and S7). The $\Delta_{c}u^{o}$ (cr)‐values were measured for each compound in a series of five or six experiments (Table 5). These $\Delta_{c}u^{o}$ (cr)‐values were used to calculate the experimental standard molar enthalpies of combustion, $\Delta_{c}H_{m}^{o}$ , (Table 5) using the following reaction:
(6)

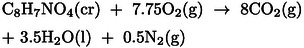




**Table 5:** Results for combustion experiments at *T*=298 K (*p°*=0.1 MPa) of the methyl‐nitrobenzoic acids.

Compound	*n* ^a^	$\Delta_{c}u^{o}$ (cr) ^b^	$\Delta_{c}H_{m}^{o}$ (cr) ^c^	$\Delta_{f}H_{m}^{o}$ (cr) ^c^
		J ⋅ g^−1^	kJ⋅ mol^−1^	kJ⋅ mol^−1^
3‐methyl‐2‐nitrobenzoic acid	5	−20487.0±3.3	−3709.3±1.5	−439.2±1.8
4‐methyl‐2‐nitrobenzoic acid	5	−20492.6±3.3	−3710.3±1.5	−438.2±1.8
5‐methyl‐2‐nitrobenzoic acid	6	−20482.7±4.1	−3708.5±1.7	−440.0±2.0
6‐methyl‐2‐nitrobenzoic acid	5	−20519.2±4.2	−3715.1±1.8	−433.4±2.1
2‐methyl‐3‐nitrobenzoic acid	6	−20464.8±4.6	−3705.2±1.9	−443.3±2.2
4‐methyl‐3‐nitrobenzoic acid	6	−20392.2±4.3	−3692.1±1.8	−456.4±2.1
6‐methyl‐3‐nitrobenzoic acid	5	−20408.5±5.1	−3695.0±2.0	−453.5±2.3
2‐methyl‐4‐nitrobenzoic acid	6	−20438.1±6.4	−3700.4±2.5	−448.1±2.7
3‐methyl‐4‐nitrobenzoic acid	6	−20377.8±3.8	−3689.5±1.6	−459.0±1.9

^a^ Number of combustion experiments. ^b^ Uncertainty of combustion energy is expressed as standard deviation of the mean. ^c^ Uncertainties are expressed as twice the standard deviation of the mean.

The standard molar enthalpies of formation in the crystalline phase, $\Delta_{f}H_{m}^{o}$ (cr), (Table 5) were calculated based on the $\Delta_{c}H_{m}^{o}$ −values of the reactions according to Eq. (6) and using Hess's Law supported by the enthalpies of formation of the reaction products H_2_O_(l)_ (−285.83 ± 0.04 kJ mol^−1^) and CO_2(g)_ (−393.51 ± 0.13 kJ mol^−1^) as assigned by CODATA.^[23]^

### Step IV. Gas‐Phase Standard Molar Enthalpies of Formation: Experimental and Computational Studies

The experimental gas‐phase standard molar enthalpies of formation $\Delta_{f}H_{m}^{o}$ (g)_exp_ are derived from the experimental enthalpies of sublimation and the results from combustion calorimetry (Table 5) according to the following thermochemical equation: 
(7)

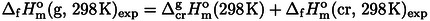




Results derived with Eq. (7) are listed in Table 6, column 4. Because the thermochemical data of the crystalline methyl‐nitrobenzoic acids were measured for the first time, computational methods are used to validate experimental results obtained in this work. Over the last decade, composite quantum chemical methods have emerged as a valuable tool for calculating *theoretical* $\Delta_{f}H_{m}^{o}$ (g, 298) values with “chemical accuracy”^[24]^ at the level of 4‐5 kJ ⋅ mol^−1^. A mismatch or match between the theoretical and experimental $\Delta_{f}H_{m}^{o}$ (g, 298 K)−values provides valuable evidence for mutual consistency or inconsistency. The most stable conformers for the methyl‐nitrobenzoic acids were located using CREST^[25]^ and further optimised using the B3LYP/6‐31g(d,p) method.^[26]^ The structures of the most stable conformers for all 10 isomers, calculated using the G3MP2^[27]^ method, are listed in Table 7.

**Table 6:** Compilation of thermochemical results at *T*=298 K (*p°*=0.1 MPa) of the methyl‐nitrobenzoic acids (in kJ mol^−1^).^a^

Compound	$\Delta_{f}H_{m}^{o}$ (cr)_exp_^b^	$\Delta_{cr}^{g}H_{m}^{o}$ ^c^	$\Delta_{f}H_{m}^{o}$ (g)_exp_	$\Delta_{f}H_{m}^{o}$ (g)_QC_^d^	Δ ^e^
3‐methyl‐2‐nitrobenzoic acid	−439.2±1.8	125.7±1.0	−313.5±2.1	−317.6±2.0	4.1
4‐methyl‐2‐nitrobenzoic acid	−438.2±1.8	121.3±1.5	−316.9±2.3	−317.7±3.5	0.8
5‐methyl‐2‐nitrobenzoic acid	−440.0±2.0	121.7±1.3	−318.3±2.4	−318.2±2.0	−0.1
6‐methyl‐2‐nitrobenzoic acid	−433.4±2.1	120.1±1.2	−313.3±2.4	−315.3±2.0	2.0
2‐methyl‐3‐nitrobenzoic acid	−443.3±2.2	121.1±1.0	−322.2±2.4	−318.7±2.0	−3.5
4‐methyl‐3‐nitrobenzoic acid	−456.4±2.1	121.1±1.0	−335.3±2.3	−334.0±2.0	−1.3
6‐methyl‐3‐nitrobenzoic acid	−453.5±2.3	120.3±1.3	−333.2±2.6	−335.4±3.5	2.2
2‐methyl‐4‐nitrobenzoic acid	−448.1±2.7	113.5±1.4	−334.6±3.0	−333.1±3.5	−1.5
3‐methyl‐4‐nitrobenzoic acid	−459.0±1.9	122.2±0.9	−336.8±2.1	−332.8±2.0	−4.0

^a^ The uncertainties are given as twice the standard deviation. ^b^ From Table 5, last column. ^c^ The evaluated values from Table 1. ^d^ From Table 8, last column. ^e^ Difference between the values reported in column 4 and 5 in this table.

**Table 7:** Structures of the most stable conformers of methyl‐nitrobenzoic acids.

Front view	Top view
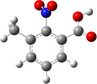	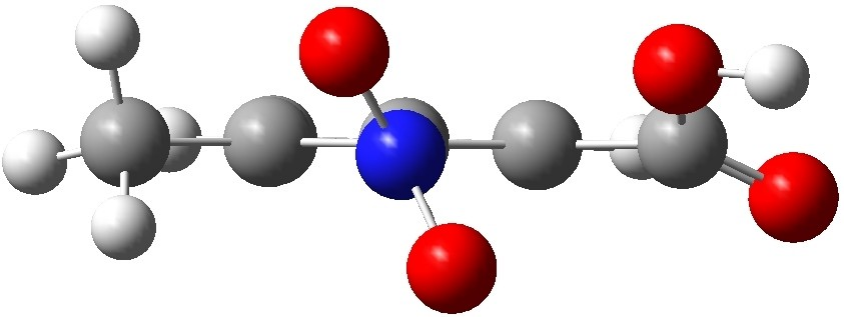
3‐methyl‐2‐nitrobenzoic acid, CAS 5437‐38‐7	
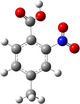	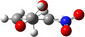
4‐methyl‐2‐nitrobenzoic acid, CAS 27329‐27‐7	
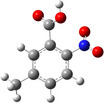	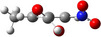
5‐methyl‐2‐nitrobenzoic acid, CAS 3113‐72‐2	
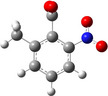	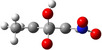
6‐methyl‐2‐nitrobenzoic acid, CAS 13506‐76‐8	
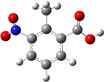	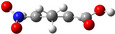
2‐methyl‐3‐nitrobenzoic acid, CAS 1975‐50‐4	
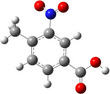	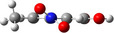
4‐methyl‐3‐nitrobenzoic acid, CAS 96‐98‐0	
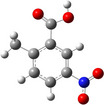	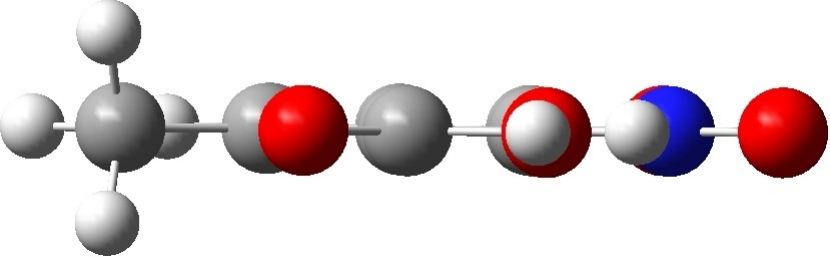
6‐methyl‐3‐nitrobenzoic acid, CAS 1975‐52‐6	
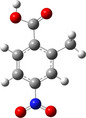	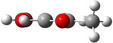
2‐methyl‐4‐nitrobenzoic acid, CAS 1975‐51‐5	
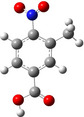	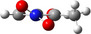
3‐methyl‐4‐nitrobenzoic acid, CAS 3113‐71‐1	
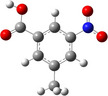	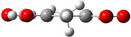

3‐methyl‐5‐nitrobenzoic acid, CAS 113882‐33‐0	

**Figure 5:**
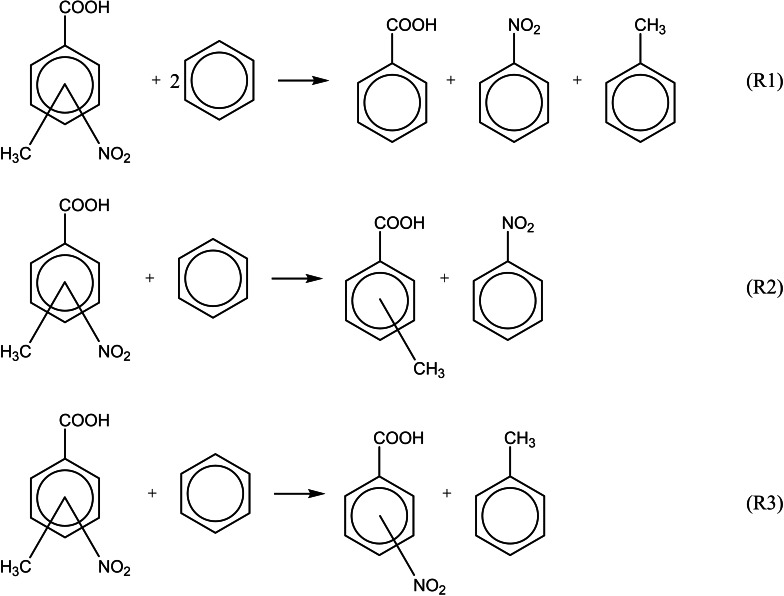
The well‐balanced reaction (WBR) used to derive the gas‐phase standard molar enthalpies of formation from the enthalpies *H*_298_ of the most stable conformers.

The enthalpies, *H*_298_, of the most stable conformers for each isomer (Table 7) were calculated using the G3MP2^[27]^ and G4^[28]^ methods and converted to the gas‐phase standard molar enthalpies of formation using the well‐balanced reactions (Figure 5).

Using these reactions and the experimental gas‐phase enthalpies of formation $\Delta_{f}H_{m}^{o}$ (g, 298 K) of benzene, toluene, nitrobenzene, methyl‐benzoic acids and nitrobenzoic acids from Table S5, the theoretical enthalpies of formation of methyl‐nitrobenzoic acids were estimated and are summarized in Table 8.

**Table 8:** The gas‐phase enthalpies of formation $\Delta_{f}H_{m}^{o}$ (g) at T=298 K (p°=0.1 MPa) for methyl‐nitrobenzoic acids, calculated with the G3MP2 and G4 methods (in kJ ⋅ mol^−1^).

	$\Delta_{f}H_{m}^{o}$ (G3MP2)_R1_^a^	$\Delta_{f}H_{m}^{o}$ (G3MP2)_R2_^b^	$\Delta_{f}H_{m}^{o}$ (G3MP2)_R3_^c^	$\Delta_{f}H_{m}^{o}$ (G4)_R1_^d^	$\Delta_{f}H_{m}^{o}$ (g)_QC_^e^
3‐methyl‐2‐nitrobenzoic acid	−317.0	−318.2	−317.0	−318.0	−317.6±2.0
4‐methyl‐2‐nitrobenzoic acid	‐	‐	‐	−317.7	−317.7±3.5
5‐methyl‐2‐nitrobenzoic acid	−317.1	−318.3	−319.9	−317.6	−318.2±2.0
6‐methyl‐2‐nitrobenzoic acid	−316.0	−314.9	−313.6	−316.4	−315.3±2.0
2‐methyl‐3‐nitrobenzoic acid	−319.8	−318.6	−318.9	−317.9	−318.7±2.0
4‐methyl‐3‐nitrobenzoic acid	−334.9	−335.0	−334.0	−332.7	−334.0±2.0
6‐methyl‐3‐nitrobenzoic acid	‐	‐	‐	−335.4	−335.4±3.5
2‐methyl‐4‐nitrobenzoic acid	‐	‐	‐	−333.1	−333.1±3.5
3‐methyl‐4‐nitrobenzoic acid	−333.1	−334.3	−332.1	−332.1	−332.8±2.0
5‐methyl‐3‐nitrobenzoic acid	‐	‐	‐	−341.3	−341.3±3.5

^a^ Calculated according to the reaction (R1). ^b^ Calculated according to the reaction (R2). ^c^ Calculated according to the reaction (R3). ^d^ Calculated according to the reaction (R1). ^e^ The weighted average value from columns 2 to 5.

The results of the G3MP2 and G4 calculations carried out with the reactions shown in Figure 5 are practically indistinguishable when considering the expanded uncertainties of ± 4.1 kJ ⋅ mol^−1^ attributed to the G3MP2 method^[27]^ and ± 3.5 kJ ⋅ mol^−1^ attributed to the G4 method.^[28]^ For this reason, we averaged the theoretical values for each MNBA and the final theoretical gas‐phase enthalpies of formation, $\Delta_{f}H_{m}^{o}$ (g)_QC_, are given in the last column of Table 8. These results are compared with the experimental values in Table 6. The agreement between the experimental and theoretical standard molar enthalpies of formation for all isomers is within the experimental uncertainties and can be considered as proof of the consistency of the thermochemical results presented in this work (Tables 1, 2 and 5). Which can now be recommended for further thermochemical calculations with substituted benzene derivatives.

### Step V. Are the Pairwise Interactions of Substituents in Tri‐Substituted Methyl‐Nitro‐Benzoic Acids Still Valid?

**Figure 6:**
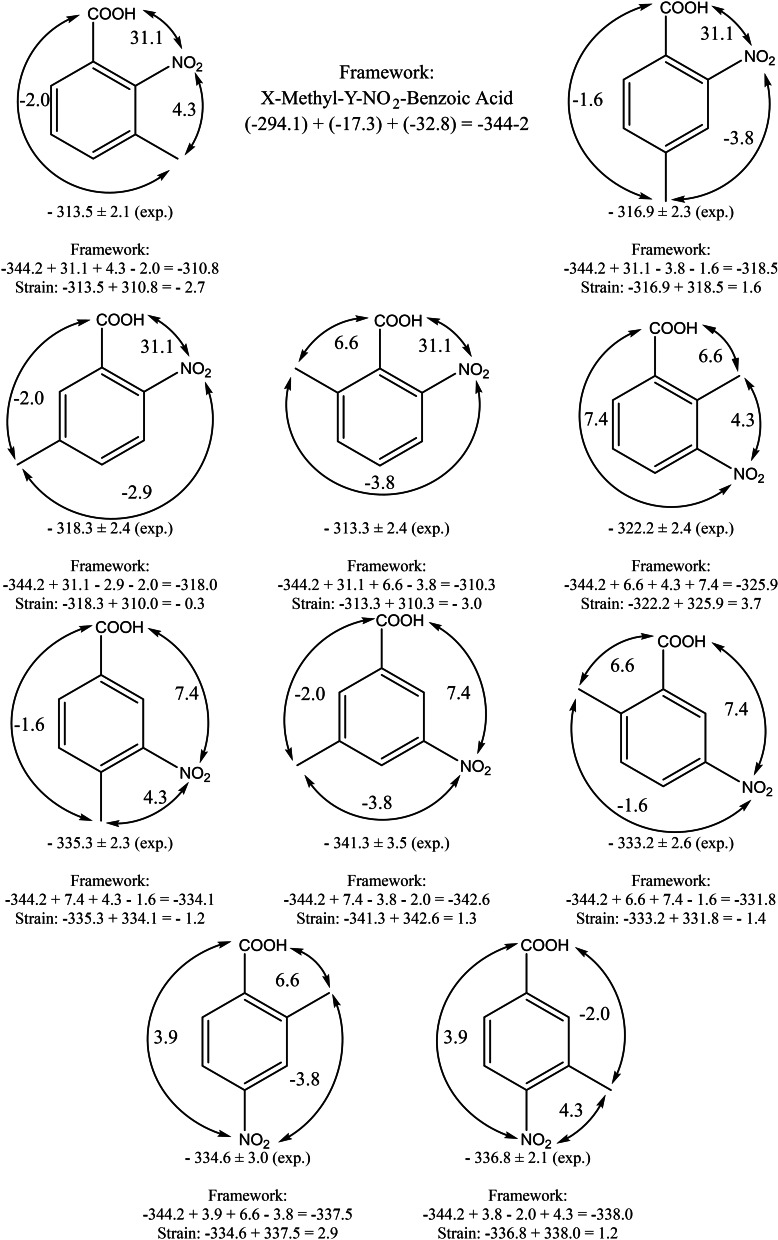
Calculation the gas‐phase standard molar enthalpies of formation, $\Delta_{f}H_{m}^{o}$ (g, 298 K), of MNBAs using benzoic acid as the “centrepiece” and quantification of the possible additional enthalpic contributions for the interactions of methyl, nitro, and carboxyl substituents, other than the pairwise interactions included in Table 4. All increments and pairwise interactions (in kJ⋅mol^−1^) are taken from Table 4.

From a physical‐organic‐chemical point of view, benzene derivatives are one of the most attractive classes of molecules. The energetics of pairwise interactions of the substituents in the *ortho*, *meta* or *para* position of the benzene ring are unique and determine the yield of the desired isomer. The pairwise interactions for methyl, nitro and carboxyl substituents are quantified accurately in this work (Table 4). It can be assumed that the introduction of the third substituent on the benzene ring increases the intensity of the mutual interactions of the substituents compared to two substituents. How strong are these additional interactions due to the perturbation in the electron density within the crowded benzene ring? Does the energetics of these interactions add up with the growing agglomeration of substituents? Can we just summarise the individual contributions? Or are there additional effects and specific interactions that we need to quantify to predict the energetics of trisubstituted benzenes? To get the answer in terms of $\Delta_{f}H_{m}^{o}$ (g), we calculated the “theoretical framework” for each isomer of methyl‐nitrobenzoic acid as shown in Figure 6.

The difference between the $\Delta_{f}H_{m}^{o}$ (g) of the “real” molecule and the gas‐phase enthalpy of formation of its model represented by the “theoretical framework” (see Figure 6) is usually referred as the “strain” (*H_S_*)[^29^, ^30^] and provides the total amount of the nearest and non‐nearest neighbour interactions (stabilising or destabilising) of substituents on the “centerpieces” with respect to $\Delta_{f}H_{m}^{o}$ (g). These differences are given in Table 9.

**Table 9:** Results for the calculations of the strain in the methyl‐nitrobenzoic acids (at *T*=298 K, *p°*=0.1 MPa, in kJ mol^−1^) ^a^

Compound	$\Delta_{f}H_{m}^{o}$ (g)_exp_^b^	$\Delta_{f}H_{m}^{o}$ (g)_CP_^c^	Δ ^d^
3‐methyl‐2‐nitrobenzoic acid	−313.5±2.1	−310.8	*−2.7*
4‐methyl‐2‐nitrobenzoic acid	−316.9±2.3	−318.5	1.6
5‐methyl‐2‐nitrobenzoic acid	−318.3±2.4	−318.0	−0.3
6‐methyl‐2‐nitrobenzoic acid	−313.3±2.4	−310.3	*−3.0*
2‐methyl‐3‐nitrobenzoic acid	−322.2±2.4	−325.9	*3.7*
4‐methyl‐3‐nitrobenzoic acid	−335.3±2.3	−334.1	−1.2
6‐methyl‐3‐nitrobenzoic acid	−333.2±2.6	−331.8	−1.4
2‐methyl‐4‐nitrobenzoic acid	−334.6±3.0	−337.5	2.9
3‐methyl‐4‐nitrobenzoic acid	−336.8±2.1	−338.0	1.2

^a^ The uncertainties are given as the twice standard deviation. In the compounds shown in italics, the possibility of the “buttress” effects of the substituents is assumed. ^b^ From Table 6, column 4. ^c^ From Figure 6 (the sum of parameters and pairwise interactions given in Table 4). ^d^ Difference between column 2 and 3 in this table, referred as the “strain” (*Hs* as shown in Figures 6 and 7) or the total amount of the nearest and non‐nearest neighbour interactions of substituents on the “centerpiece” (benzoic acid in this table).

The actual amount of interactions in methyl‐nitrobenzoic acids is given in Table 9, column 4 and is comparable (within the uncertainties associated with $\Delta_{f}H_{m}^{o}$ (g)_exp_‐values) to the sum of parameters, nearest and non‐nearest neighbour interactions of substituents (Table 9, column 3). This observation indicates that no additional strain arises for the tri‐substituted (CH_3_, NO_2_, COOH) benzenes if the energetics is calculated from the sum of the pairwise interactions from Table 4. Surprisingly, even for the 1,2,3‐substituted molecules (3‐methyl‐2‐nitrobenzoic acid, 6‐methyl‐2‐nitrobenzoic acid, and 2‐methyl‐3‐nitrobenzoic acid) no noticeable additional strain (within the experimental uncertainties) is inherent for these molecules. On one hand, this observation answers the question posed in the title of this paper. On the other hand, this contradicts our expectations and experiences with the tri‐substituted (CH_3_O, CH_3_O, CHO) benzenes, in which, for example, 2,6‐dimethoxybenzaldehyde was additionally strained by 8.8 kJ mol^−1[31]^ due to the “buttress” effect of three bulky substituents. By contrast, 3‐methyl‐2‐nitrobenzoic acid and 6‐methyl‐2‐nitrobenzoic acid appear to be slightly ‘stabilised’, at ≈−3 kJ mol^−1^, as shown in Table 9, column 4. However, 2‐methyl‐3‐nitrobenzoic acid is additionally “strained” by 3.7 kJ mol^−1^, which corresponds to our expectations. Even if these 1,2,3‐additional interactions only slightly overwhelm the experimental uncertainties, it is interesting to analyse the concurrent orientations of the CH_3_, NO_2_ and COOH substituents, particularly in the 1,2,3‐sequence in the benzene ring, to see if their screw relative to the benzene ring differs from the orientations in the di‐substituted benzenes.

As can be seen from Table S10, the substituents on the di‐substituted benzenes are strictly in the same plane as the benzene ring, regardless of whether the nitro and carboxyl groups are in the meta‐ or para‐position to one another on the benzene ring. In contrast, for the ortho‐substituted benzenes, it is expected that the interactions of the nearest neighbour will twist the substituents out of the plane of the benzene ring, in order to possibly avoid steric repulsion. However, in 2‐methyl‐nitrobenzoic acid, the carboxylic acid substituent is still in the same plane as the benzene ring, but due to steric repulsion, it causes a moderate strain of 6.6 kJ mol^−1^ (Figure S3). To avoid steric repulsion, the nitro group in 2‐methylnitrobenzene rotates 34.4 ° relative to the plane of the benzene ring, and the resulting strain for this molecule is only 4.3 kJ mol^−1^ (Figure S3). In 2‐nitro‐benzoic acid, both substituents are forced out of the plane: the nitro group is rotated by 38.7 ° and the carboxyl group by 45.7 °, but since both groups are too bulky, the molecule remains strained by 31.1 kJ mol^−1^ (Figure S3). These individual levels of strains in the di‐substituted benzenes correspond to the pairwise interactions quantified in Table 4.

**Figure 7:**
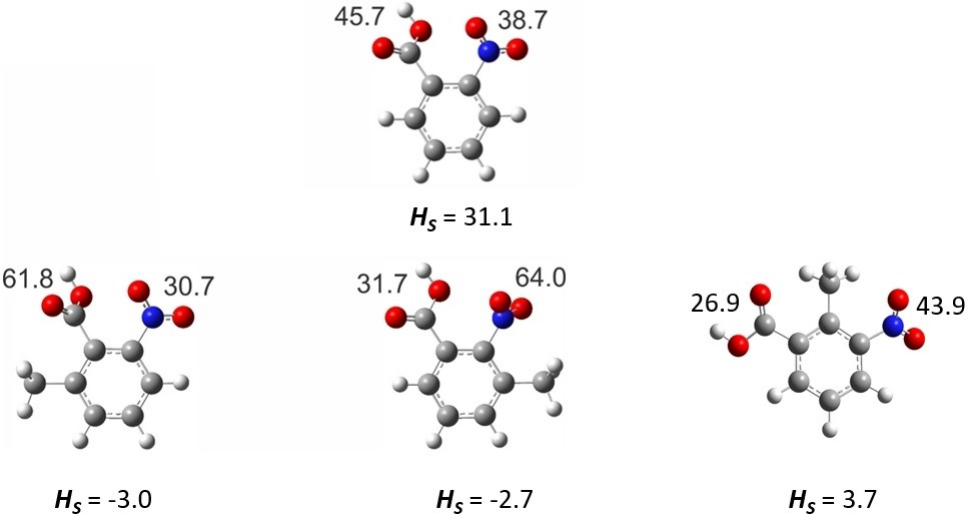
Comparison of the skewing of the carboxy and nitro substituents in ortho‐substituted benzenes with respect to the plane of the benzene ring (the torsion angles are given in degrees) and comparison of the strain values, *H_S_*, (in kJ⋅mol^−1^), taken from Table 9.

In the tri‐substituted methyl‐nitrobenzoic acids, the nitro group only retains its planar position relative to the ring plane if there are no neighbouring substituents in its close proximity. The carboxyl group, which is isolated from the nearest neighbouring substituents, also retains its orientation in the plane of the benzene ring. Comparison of the skewing of the carboxy and nitro substituents in ortho‐substituted benzenes with respect to the plane of the benzene ring is shown in Figure 7. It is interesting to note, that the presence of a methyl group in the ortho‐position in benzoic acid does not affect the carboxyl orientation when the nitro group is in the para position, as in 2‐methyl‐4‐nitrobenzoic acid (Table S11), or the methyl is in the ortho‐position, as in 6‐methyl‐3‐nitrobenzoic acid. However, for 2‐methyl‐3‐nitrobenzoic acid, in which the methyl group is located between the nitro and carboxyl groups, the strain that arises in this case forces the carboxyl group to leave its planar position and rotate through an angle of 26.9 °.

From an energetic point of view, the more planar the substituent is with the benzene ring, the greater the stability of the molecule. The 1,2‐ and 1,2,3‐positions of the substituents on the benzene ring distort the planarity of the molecule. Therefore, the driving force for possible molecular stabilisation is the interplay of the torsion angles of the substituents, as shown in Figure 7. Therefore, the methyl group in the ortho‐position in 6‐methyl‐2‐nitrobenzoic acid and in 3‐methyl‐2‐nitrobenzoic acid disturbs the torsion angles of the carboxyl and nitro group (in comparison to 2‐nitrobenzoic acid) and even stabilises these two molecules slightly. In contrast, 2‐methyl‐3‐nitrobenzoic acid is slightly destabilised. One possible reason for the latter effect is that the carboxyl group in this molecule generates repulsions with the CH_3_ group through the carbonyl function (and not through the hydroxyl group as in 6‐methyl‐2‐nitrobenzoic acid and 3‐methyl‐2‐nitrobenzoic acid). Perhaps in the case of 2‐methyl‐3‐nitrobenzoic acid, there are more spatial restrictions on the 1,2,3 sequence of substituents and the molecule is finally destabilised.

## Conclusion

A comprehensive study of the three main phase transitions of nine methyl‐substituted nitrobenzoic acids was carried out using a variety of techniques and is presented in this article. A consistent series of phase transition enthalpies was determined, evaluated, and is reported.

The temperature dependencies of the vapour pressures of the high‐purity samples were determined using the classic Knudsen effusion mass loss method and transpiration method. The standard molar sublimation enthalpies were derived from these measurements and are important for quantifying the strength of intermolecular interactions.

DSC was used to investigate the thermal behaviour of the samples and to determine the melting temperatures and fusion enthalpies. With the exception of 6‐methyl‐2‐nitrobenzoic acid, no solid‐solid phase transitions were observed in the samples studied prior to melting. It was observed that the samples with the substituent in the ortho positions exhibit higher fusion enthalpies compared to meta‐ and para‐substituted molecules. The relative phase stability of the ortho‐substituted samples deserves more attention to explain this phenomenon.

A ‘“centerpiece” approach has been developed for estimating the enthalpies of vaporisation of benzoic acid derivatives. This approach was validated using experimental data derived from sublimation and fusion enthalpies. Additional support for the “centerpiece” approach was obtained by directly determining the enthalpies of vaporisation using non‐isothermal thermogravimetry.

Combustion calorimetry was used to measure the standard molar enthalpies of formation of methyl nitrobenzoic acids in the crystalline phase. These values, in combination with the evaluated sublimation enthalpies, provide their standard molar enthalpies of formation in the gas phase. High‐level quantum chemical methods were used to cross‐validate the theoretical and experimental gas‐phase enthalpies of formation. These results were used to extend the “centerpiece” approach to the estimation of gas‐phase enthalpies of formation and to derive and analyse the pairwise interactions of the substituents on the benzene ring. It turns out that only the sum of pairwise interactions (derived from the di‐substituted benzenes) is sufficient to predict the gas‐phase formation enthalpies of the tri‐substituted benzenes (with methyl, nitro and carboxyl substituents). The investigation of tri‐substituted benzenes with substituents other than these three is the subject of a forthcoming work.

## Experimental Section

### Thermochemical Methods

The samples of methyl‐nitrobenzoic acids (MNBAs) used in this work were of commercial origin (see Table S12). The samples for combustion calorimetry, Knudsen and transpiration experiments were purified using fractional sublimation under reduced pressure The final purities of the samples were determined by DSC resulting in mole fractions in the range of 0.989 to 0.999 as reported in the supplementary information.

Before starting the vapour pressure measurements, the samples were preconditioned in the transpiration saturator or in the Knudsen cell at 373 K in order to remove possible traces of volatile impurities. The small residual amount of impurities found in the samples for the thermochemical studies belong to the isomeric methylnitrobenzoic acids with very similar thermodynamic properties, so that the traces of these isomers cannot in principle have any effect on the results for the main component.

The absolute vapour pressures at different temperatures above the solid samples were measured using the Knudsen effusion mass loss method and transpiration method. The standard molar enthalpies of sublimation were derived from the temperature dependence of the vapour pressures. The standard molar enthalpies of vaporisation were derived from the temperature dependence of the mass‐loss rates measured using the non‐isothermal thermogravimetry. The melting temperatures and the standard molar enthalpies of fusion were measured by DSC. A concise description of the experimental methods and the required details are given in ESI.

### Computational Methods

The software Gaussian 16 series^[32]^ was used for the quantum chemical calculations. The stable conformers of each compound were found using a computer program CREST (conformer‐rotamer ensemble sampling tool)^[25]^ and their total *H*_298_‐values were calculated using the G3MP2^[27]^ and G4 methods^[28]^ under assumption “rigid rotator‐harmonic oscillator”. The *H*_298_‐values were finally converted to the gas‐phase standard molar enthalpies, $\Delta_{f}H_{m}^{o}$ (g, 298 K)_QC_, and discussed. The details on computation can be found elsewhere.^[33]^

## Conflict of Interests

The authors declare no conflict of interest.

## Supporting information

As a service to our authors and readers, this journal provides supporting information supplied by the authors. Such materials are peer reviewed and may be re‐organized for online delivery, but are not copy‐edited or typeset. Technical support issues arising from supporting information (other than missing files) should be addressed to the authors.

Supporting Information
