# Supplementary material for: Thermodynamic Insights on the Structure‐Property Relationships in Substituted Benzenes: Are the Pairwise Interactions in Tri‐Substituted Methyl‐Nitro‐Benzoic Acids Still Valid?
Source: Chempluschem. 2025 Feb 11;90(5):e202400703. doi: 10.1002/cplu.202400703 (PMC12105461; doi:10.1002/cplu.202400703)
Supplement: Supplementary file 1 — Supporting Information [file CPLU-90-e202400703-s001.pdf]

ChemPlusChem

Supporting Information

Thermodynamic Insights on the Structure-Property Relationships in Substituted Benzenes: Are the Pairwise Interactions in Tri-Substituted Methyl-Nitro-Benzoic Acids Still Valid?

José M. Silva Ferraz,* Vladimir N. Emel'yanenko, Dzmitry H. Zaitsau, Artemiy A. Samarov, Bruno Brunetti, Andrea Ciccioli, Stefano Vecchio Ciprioti, and Sergey P. Verevkin*

ELECTRONIC SUPPORTING INFORMATION

Thermodynamic Insights on the Structure-Property Relationships in Substituted Benzenes: Are the Pairwise Interactions in Tri-Substituted Methyl-Nitro-Benzoic Acids Still Valid?

José M. Silva Ferraz [a],*, Vladimir N. Emel´yanenko[b], Dzmitry H. Zaitsau [c,d], Artemiy A. Samarov [e], Bruno Brunetti[f], Andrea Ciccioli[g], Stefano Vecchio Ciprioti[a], Sergey P. Verevkin [b,c],*

[a] J.M. Silva Ferraz, S. Vecchio Ciprioti
Department of Basic and Applied Sciences for Engineering (S.B.A.I.)
Sapienza University of Rome
Via del Castro Laurenziano 7, Building RM017, 00161 Rome, Italy
E-mail: josemiguel.silvaferraz@uniroma1.it

[b] V.N. Emel'yanenko, S.P. Verevkin
Competence Centre CALOR, Department Life, Light & Matter
Faculty of Interdisciplinary Research, University of Rostock
18059 Rostock, Germany
E-mail: sergey.verevkin@uni-rostock.de

[c] D.H. Zaitsau, S.P. Verevkin
Institute of Technical Thermodynamics
University of Rostock
18059 Rostock, Germany

[d] D.H. Zaitsau
FVTR GmbH
18059 Rostock, Germany

[e] A.A. Samarov
Saint Petersburg State University
Peterhof, 198504 Saint Petersburg, Russian Federation

[f] B. Brunetti
Istituto per lo Studio dei Materiali Nanostrutturati, Consiglio Nazionale delle Ricerche, Department of Chemistry
Sapienza University of Rome
P.le Aldo Moro 5, 00185, Rome, Italy

[g] A. Ciccioli
Department of Chemistry
Sapienza University of Rome
P.le Aldo Moro 5, 00185, Rome, Italy

Thermodynamic workflow

According to textbook knowledge, three common equations relate the thermochemical properties:

$$\Delta_f H^o_m(g) = \Delta_f H^o_m(cr) + \Delta^g_{cr} H^o_m \quad (S1)$$

$$\Delta_f H^o_m(g) = \Delta_f H^o_m(liq) + \Delta^g_l H^o_m \quad (S2)$$

$$\Delta^g_{cr} H^o_m = \Delta^g_l H^o_m + \Delta^l_{cr} H^o_m \quad (S3)$$

Admittedly, the gas-phase enthalpy of formation, $\Delta_f H^o_m$(g), cannot be measured, however, it is common in physical chemistry to name the result of the summation the condensed state enthalpy of formation, $\Delta_f H^o_m$(liq or cr), with the corresponding vaporisation (or sublimation) enthalpy according to Eqs. (S1) or (2) as "experimental" enthalpy of formation. The sublimation enthalpies, $\Delta^g_{cr} H^o_m$, and vaporisation enthalpies, $\Delta^g_l H^o_m$, are usually measured directly calorimetrically or derived from the vapour pressure temperature dependences, whereby these two phase transition enthalpies are related to each other by Eq. (S3) where $\Delta^l_{cr} H^o_m$ is the standard molar enthalpy of fusion, easily measurable using differential scanning calorimetry (DSC). In thermochemistry, it is common to adjust all enthalpies involved in Eqs. (S1) - (S3) to an arbitrary but common reference temperature. In this work we have chosen T = 298.15 K as the reference temperature.

There is a long tradition in science of relying on experimental results rather than empirical or theoretical knowledge. Authors follow this tradition unreservedly. However, all experimental enthalpies involved in Eq. (S1) – (S3) could be affected by occasional or systematic errors due to equipment defects or malfunctions or insufficient sample purity. For this reason, any single value available in the literature should be considered suspect until some sort of validation is performed on that value. The straightforward validation consists in repeating the enthalpy measurement (preferably with a different technique). The agreement of old and new results is the best way to dispel a suspicion. But what to do if the results are different? Which result is preferred? In our experience, the most recent result is most used for chemical-engineering calculations. Is that right? Obviously not, as both results must be checked for consistency with the entire network of experimental thermochemical properties already available. Only after the test has been passed can the old or new value be regarded as reliable. In this context, the development of diagnostic tools for validation of experimental data is in the main task of our recent studies.

Structure-property relationships in physical chemistry are the most recognized empirical tool to test and establish useful regularities within the set of structurally similar molecules. These relationships are quite important from an educational point of view, as they facilitate the understanding of the available data. In addition, they also allow a reasonable prediction of properties that have not yet been studied.

Over the last decade, composite quantum chemical methods have emerged as a valuable tool for calculating *theoretical* $\Delta_f H^o_m$(g, 298.15)-values with "chemical accuracy" at the level of 4-5 $kJ \cdot mol^{-1}$. An agreement or disagreement between the *theoretical* and *experimental* $\Delta_f H^o_m$(g, 298.15 K)-values provides valuable evidence for the data mutual consistency. The latter property is linked to the condensed state properties by the Eqs. S1 and S2 and opens an independent way for mutual validation of the $\Delta_f H^o_m$(liq or cr)-values and the enthalpies of phase transitions, $\Delta^g_{cr} H^o_m$, and $\Delta^g_l H^o_m$.

Therefore, essentially the main purpose of this work, is to show that the combination of QC methods with structure-property relationships and with group additivity methodology provides a reliable diagnostic of the quality of thermochemical data for any organic molecule of interest. Since this combination encompasses the computational algorithms from simple least squares treatment of the data matrices to the high-level quantum chemical calculations, we refer to this combination as "in silico" assisted diagnostics of available enthalpy data involved in Eqs. (S1) - (S3). This diagnostic, comprises a few steps.

Step I: in the first step, the diagnostics of the phase transitions is performed. The available sublimation, $\Delta^g_{cr} H^o_m$ (298.15 K), and vaporisation enthalpies, $\Delta^g_l H^o_m$ (298.15 K) for a series of

molecules of interest are collected and analysed. Depending on the quantity and quality of the available data, complementary measurements may be carried out to resolve contradictions between the available data. In fact, of the enthalpies of phase transitions (liquid-gas, crystal-gas and crystal-liquid), only the enthalpy of vaporization obeys the additive rules and structure-property relationships and can be easily validated by these methods. However, the Eq. S3 enables the phase transition data to be interrelated and the enthalpies of vaporization to be derived in order to assess their quality.

Step II: in the second step, the solid-liquid phase transitions studied using the conventional DSC technique and the resulting enthalpies of fusion are used to reconcile the solid-gas and liquid gas phase transition enthalpies.

Step III: in the third step, the diagnostics of the condensed state enthalpies of formation is conducted. The available data on $\Delta_f H^o_m$(liq or cr) are collected and analysed to make a decision on the need to carry out the complementary measurements. The evaluated enthalpies of formation are combined with the enthalpies of sublimation and enthalpies of vaporization evaluated in Step I to produce the set of experimental gas-phase enthalpies of formation according to equations (S1) and (S2) for validation of the quantum-chemical calculations.

Step IV: in step four, the "in silico" diagnostics is continued by using Eqs. (1) and (2). For this purpose, few high-level G*-family composite quantum-chemical (QC) methods (*e.g.* G3MP2 and G4 methods) are used to derive the theoretical gas-phase enthalpies of formation, $\Delta_f H^o_m$(g, 298.15 K), for several similarly shaped molecules for which reliable experimental data are available. Usually, the chosen QC-methods agree well with the experiment and validate the calculations for the desired subclass of molecules and in particular the "theoretical" $\Delta_f H^o_m$(g, 298.15 K)-value for the molecule of interest. Once the consistent and reliable values of the enthalpies of formation have been determined, they are used to better understand the energetics of molecules and their structure-property relationships.

Step V: in step five, the validated $\Delta_f H^o_m$(g, 298.15 K)-values for ten methyl nitrobenzoic acid isomers are used to analyse the energetics of substituent accumulation on the benzene ring and to explore the limits of group additivity for the mono-, di-, and tri-substituted benzenes.

Knudsen Effusion Mass Loss Method

The Knudsen effusion mass loss (KEML) experiments were carried out using a Ugine-Eyraud Model B60 Setaram thermobalance, accurately described in a previous paper [34]. It is essentially constituted by a furnace, a microbalance and a vacuum system. The measuring cell is housed in a copper cylinder with a cap, which has the purpose of equalizing the temperature of the sample to allow an optimal temperature measurement. The copper cylinder is suspended to the arm of the microbalance with a standard measurement uncertainty u(m) = 0.01 mg. The temperature was measured via a Pt100 Platinum Resistance Thermometer inserted into the copper cylinder, being the standard measurements uncertainty u(T) less than 0.2 K. The temperature control and the measurements of the mass loss are made through a data logger (HP 34970A) driven by a LabVIEW software that permits the continuous control of the system.

Three alumina cells with different effusion orifice diameters (OD) of 3, 1, and 0.2 mm were alternatively used by loading approximately 50 mg of sample. The instrumental Knudsen constant[35] was evaluated for each cell by performing KEML experiments under identical conditions of the compounds tested using very pure calibration substances having well known vapor pressures (benzoic acid[36] in this study). During each KEML experiment the temperature of the sample was adjusted to evaluate the mass loss rate at different constant temperatures. For each experiment, the isothermal temperature explored was first decreased and then increased. This approach allows detecting possible changes in the composition of the sample caused by impurities or decomposition reactions. This procedure would produce a gradual variation of the sample's vapor pressures, and, therefore, two non-overlapping data sets.

The vapor pressures, p_i, of MNBAs measured at different temperatures T were approximated by the following equation:

$$R \cdot \ln(p_\mathrm{i}/p_\mathrm{ref}) = \mathrm{a} + \frac{\mathrm{b}}{T} + \Delta_\mathrm{cr}^\mathrm{g} C_\mathrm{p,m}^\mathrm{o} \cdot \ln\left(\frac{T}{T_0}\right) \quad \text{(S4)}$$

where a and b are adjustable parameters, the arbitrary temperature T_0 = 298.15 K, R = 8.314462 J·K^{-1}·mol^{-1} [37]is the molar gas constant, the reference pressure $p_\mathrm{ref} = 1$ Pa, and $\Delta_\mathrm{cr}^\mathrm{g} C_\mathrm{p,m}^\mathrm{o} = C_\mathrm{p,m}^\mathrm{o}(\mathrm{g}) - C_\mathrm{p,m}^\mathrm{o}(\mathrm{cr})$ is the difference between the standard molar heat capacities of the gaseous $C_\mathrm{p,m}^\mathrm{o}(\mathrm{g})$ and the crystalline phase $C_\mathrm{p,m}^\mathrm{o}(\mathrm{cr})$, respectively.
The standard molar enthalpies of sublimation of MNBAs at temperatures T were derived from the temperature dependence of vapor pressures approximated by Eq. (S4) using the following equation:

$$\Delta_\mathrm{cr}^\mathrm{g} H_\mathrm{m}^\mathrm{o}(T) = -b + \Delta_\mathrm{cr}^\mathrm{g} C_{p,\mathrm{m}}^\mathrm{o} \times T \quad \text{(S5)}$$

where b is one of the adjustable parameters of Eq. (S4). The standard molar sublimation entropies at temperatures T were also derived from the temperature dependences of vapor pressures (approximated by Eq. S4) as follows:

$$\Delta_\mathrm{cr}^\mathrm{g} S_\mathrm{m}^\mathrm{o}(T) = \Delta_\mathrm{cr}^\mathrm{g} H_\mathrm{m}^\mathrm{o}/T + R \times \ln(p_\mathrm{i}/p^\mathrm{o}) \quad \text{(S6)}$$

with p^o = 0.1 MPa.

The sublimation enthalpies $\Delta_{cr}^{g}H_{m}^{o}$(298.15 K) at the reference temperature T = 298.15 K of the MNBAs were calculated using Eqs. (S4) and (S5) with the $\Delta_{cr}^{g}C_{p,m}^{o}$ = -33.9 J·mol^{-1}·K^{-1} (derived from the $C_{p,m}^{o}$(cr) = 220.8 J·mol^{-1}·K^{-1}, estimated using the empirical method developed by Chickos *et al*.[38]

Table S1. Results of Knudsen effusion method for methyl-nitrobenzoic acids: absolute vapor pressures, p_i, standard (p^o = 0.1 MPa) molar sublimation enthalpies, $\Delta_{cr}^{g}H_{m}^{o}$, and standard (p^o = 0.1 MPa) molar sublimation entropies $\Delta_{cr}^{g}S_{m}^{o}$ measured in this work

T/ K[a]	m/ mg[b]	t/ s[c]	p/ Pa[d]	$u(p)$/ Pa[e]	$\Delta_{cr}^{g}H_{m}^{o}(T)$[f]/ kJ·mol^{-1}	$\Delta_{cr}^{g}S_{m}^{o}(T)$[g]/ J·K^{-1}·mol^{-1}
3-methyl-2-nitrobenzoic acid; Orifice – 0.2 mm						
$\Delta_{cr}^{g}H_{m}^{o}$(298.15 K) = 127.4 ± 3.6 kJ·mol^{-1}						
$\Delta_{cr}^{g}S_{m}^{o}$(298.15 K) = 237.6 ± 3.0 J·mol^{-1}·K^{-1}						
$\Delta_{cr}^{g}G_{m}^{o}$(298.15 K) = 56.6 ± 0.1 kJ·mol^{-1}						
$\ln(p_i/p_{ref}) = \frac{367.2}{R} - \frac{137497}{RT} - \frac{33.9}{R}\ln\frac{T}{298.15}$; p_{ref} = 1 Pa						
372.7	0.38	36899	0.32	-0.01	124.9	229.8
374.5	0.26	19399	0.41	0.01	124.8	230.1
376.5	0.56	34384	0.50	0.01	124.7	229.9
378.4	0.56	28395	0.60	0.00	124.7	229.5
380.5	0.14	6020	0.75	0.00	124.6	229.3
380.5	0.10	4101	0.74	-0.01	124.6	229.2
380.5	0.21	9286	0.72	-0.03	124.6	229.0
384.3	0.58	16772	1.10	-0.01	124.5	228.9
386.2	0.71	16611	1.35	0.01	124.4	228.9
388.2	0.53	10471	1.59	-0.04	124.3	228.4
390.1	0.65	10344	1.99	0.02	124.3	228.6
392.1	0.57	7467	2.42	0.03	124.2	228.4
394.0	0.71	7740	2.93	0.04	124.1	228.3
396.0	0.61	5440	3.57	0.09	124.1	228.2
396.1	0.64	5857	3.51	0.02	124.1	228.0
397.6	0.65	5344	3.89	-0.15	124.0	227.5
4-methyl-2-nitrobenzoic acid; Orifice – 0.2 mm						
$\Delta_{cr}^{g}H_{m}^{o}$(298.15 K) = 123.1 ± 3.6 kJ·mol^{-1}						
$\Delta_{cr}^{g}S_{m}^{o}$(298.15 K) = 233.6 ± 3.1 J·mol^{-1}·K^{-1}						
$\Delta_{cr}^{g}G_{m}^{o}$(298.15 K) = 53.8 ± 0.1 kJ·mol^{-1}						
$\ln(p_i/p_{ref}) = \frac{362.2}{R} - \frac{133235.3}{RT} - \frac{33.9}{R}\ln\frac{T}{298.15}$; p_{ref} = 1 Pa						
369.5	0.37	22434	0.51	0.01	120.7	225.4
371.5	0.43	21364	0.62	0.00	120.6	225.1
373.4	0.42	16496	0.78	0.02	120.6	225.2

379.2	0.44	10001	1.38	-0.01	120.4	224.4
381.2	0.36	6671	1.67	-0.02	120.3	224.2
383.2	0.45	7083	2.01	-0.04	120.2	223.9
385.0	0.40	5330	2.38	-0.09	120.2	223.6
387.0	0.51	5410	2.95	-0.03	120.1	223.7
389.0	0.62	5267	3.70	0.08	120.0	223.7
390.9	0.73	5243	4.43	0.09	120.0	223.6
396.7	1.41	6020	7.49	0.08	119.8	223.0

5-methyl-2-nitrobenzoic acid; Orifice – 0.2 mm
$\Delta_{cr}^{g}H_{m}^{o}$(298.15 K) = 122.9 ± 2.8 kJ·mol^{-1}
$\Delta_{cr}^{g}S_{m}^{o}$(298.15 K) = 236.4 ± 2.3 J·mol^{-1}·K^{-1}
$\Delta_{cr}^{g}G_{m}^{o}$(298.15 K) = 52.5 ± 0.1 kJ·mol^{-1}

$\ln(p_i/p_{ref}) = \frac{366.0}{R} - \frac{133052.3}{RT} - \frac{33.9}{R}\ln\frac{T}{298.15}$; p_{ref} = 1 Pa

357.4	0.39	52074	0.23	0.01	120.9	230.4
359.3	0.50	52369	0.29	0.01	120.9	230.4
361.2	0.32	28445	0.34	0.01	120.8	229.8
363.2	0.38	28084	0.42	0.01	120.7	229.5
365.1	0.34	19066	0.54	0.01	120.7	229.7
367.1	0.40	19234	0.65	0.02	120.6	229.3
369.0	0.34	13183	0.79	0.02	120.5	229.0
371.0	0.43	13089	1.00	0.02	120.5	229.1
372.9	0.39	10276	1.19	0.03	120.4	228.6
374.8	0.49	10297	1.48	0.03	120.3	228.6
376.8	0.40	7018	1.80	0.04	120.3	228.4
378.8	0.48	7064	2.11	0.04	120.2	227.9
380.6	0.47	5469	2.67	0.06	120.1	228.1
382.6	0.54	5341	3.19	0.06	120.1	227.8
384.6	0.67	5301	3.99	0.07	120.0	227.9
386.5	0.78	5314	4.66	0.08	119.9	227.4
388.4	1.02	5366	6.04	0.09	119.9	227.9
390.4	1.13	5270	6.81	0.10	119.8	227.1
392.4	2.61	9441	8.81	0.11	119.8	227.6
394.4	1.90	6128	9.87	0.13	119.7	226.8

6-methyl-2-nitrobenzoic acid; Orifice – 0.2 mm
$\Delta_{cr}^{g}H_{m}^{o}$(298.15 K) = 123.7 ± 5.6 kJ·mol^{-1}
$\Delta_{cr}^{g}S_{m}^{o}$(298.15 K) = 242.3 ± 4.6 J·mol^{-1}·K^{-1}
$\Delta_{cr}^{g}G_{m}^{o}$(298.15 K) = 51.5 ± 0.1 kJ·mol^{-1}

$\ln(p_i/p_{ref}) = \frac{371.9}{R} - \frac{133789.8}{RT} - \frac{33.9}{R}\ln\frac{T}{298.15}$; p_{ref} = 1 Pa

358.9	0.33	23273	0.43	0.01	121.6	236.1
360.9	0.43	25567	0.52	0.01	121.6	235.6

362.9	0.62	27951	0.68	0.01	121.5	235.8
364.8	0.50	19140	0.80	0.02	121.4	235.3
366.8	0.67	19599	1.04	0.02	121.4	235.5
368.7	0.44	11291	1.19	0.03	121.3	234.7
370.7	0.54	10550	1.57	0.03	121.2	235.0
372.6	0.42	7154	1.80	0.04	121.2	234.4
374.6	0.53	6924	2.37	0.05	121.1	234.7
376.5	0.62	6900	2.80	0.05	121.0	234.3
378.4	0.60	5266	3.55	0.07	121.0	234.4
380.5	0.69	5174	4.19	0.08	120.9	233.9

2-methyl-3-nitrobenzoic acid; Orifice – 0.2 mm
$\Delta_{cr}^{g}H_{m}^{o}$(298.15 K) = 121.8± 4.8 kJ·mol^{-1}
$\Delta_{cr}^{g}S_{m}^{o}$(298.15 K) = 232.2 ± 4.0 J·mol^{-1}·K^{-1}
$\Delta_{cr}^{g}G_{m}^{o}$(298.15 K) = 52.6 ± 0.1 kJ·mol^{-1}

$\ln(p_i/p_{ref}) = \frac{361.8}{R} - \frac{131935}{RT} - \frac{33.9}{R}\ln\frac{T}{298.15}$; p_{ref} = 1 Pa

360.9	0.47	49917	0.29	0.01	119.7	225.6
362.8	0.84	69997	0.37	0.01	119.6	225.7
364.8	0.52	34045	0.47	0.01	119.6	225.8
367.5	1.09	57785	0.58	0.01	119.5	224.9
368.7	0.50	23092	0.67	0.01	119.4	224.9
372.6	0.50	16188	0.97	0.02	119.3	224.2
374.5	0.59	14984	1.22	0.02	119.2	224.3
376.5	0.51	10074	1.57	0.03	119.2	224.6
378.4	0.59	10110	1.81	0.04	119.1	224.0
380.3	0.47	6756	2.19	0.05	119.0	223.8
382.4	0.60	6892	2.72	0.05	119.0	223.7
384.3	0.56	5311	3.29	0.06	118.9	223.6
386.2	0.63	4946	4.04	0.07	118.8	223.6

2-methyl-3-nitrobenzoic acid; Orifice – 1.0 mm
$\Delta_{cr}^{g}H_{m}^{o}$(298.15 K) = 123.7± 2.8 kJ·mol^{-1}
$\Delta_{cr}^{g}S_{m}^{o}$(298.15 K) = 238.6 ± 2.3 J·mol^{-1}·K^{-1}
$\Delta_{cr}^{g}G_{m}^{o}$(298.15 K) = 52.5 ± 0.1 kJ·mol^{-1}

$\ln(p_i/p_{ref}) = \frac{368.3}{R} - \frac{133798}{RT} - \frac{33.9}{R}\ln\frac{T}{298.15}$; p_{ref} = 1 Pa

329.1	0.21	24915	0.0068	0.0003	122.6	235.4
331.1	0.23	21940	0.0085	0.0004	122.6	234.9
333.0	0.52	37549	0.0112	0.0003	122.5	234.9
335.0	0.58	33531	0.0139	0.0004	122.4	234.2
337.4	0.54	21493	0.0203	0.0006	122.4	234.5
339.4	0.59	18801	0.0256	0.0007	122.3	234.1
341.3	0.76	18361	0.0338	0.0008	122.2	234.2

343.3	0.84	15928	0.0430	0.0010	122.2	233.9
345.2	0.73	10552	0.0563	0.0014	122.1	234.0
347.2	0.80	9681	0.0677	0.0016	122.0	233.3
349.1	0.81	7318	0.0907	0.0022	122.0	233.7
351.1	0.89	6713	0.1090	0.0025	121.9	233.0
353.0	0.82	4709	0.1433	0.0034	121.8	233.2
355.0	0.90	4320	0.1723	0.0040	121.8	232.6
356.9	1.57	5825	0.2242	0.0046	121.7	232.8
358.9	1.73	5344	0.2695	0.0054	121.6	232.3
360.8	2.50	6376	0.3287	0.0063	121.6	232.0
362.8	2.75	5750	0.4021	0.0076	121.5	231.6

2-methyl-3-nitrobenzoic acid; Orifice – 3.0 mm

$\Delta_{cr}^{g}H_{m}^{o}$(298.15 K) = 124.1± 4.0 kJ·mol^{-1}

$\Delta_{cr}^{g}S_{m}^{o}$(298.15 K) = 242.3 ± 2.3 J·mol^{-1}·K^{-1}

$\Delta_{cr}^{g}G_{m}^{o}$(298.15 K) = 51.9 ± 0.1 kJ·mol^{-1}

$\ln(p_i\,/p_{ref}) = \frac{371.9}{R} - \frac{134210}{RT} - \frac{33.9}{R}\ln\frac{T}{298.15}$; p_{ref} = 1 Pa

309.6	0.21	62558	0.0005	0.0001	123.7	241.3
313.5	0.26	44755	0.0009	0.0001	123.6	240.6
315.4	0.38	50801	0.0012	0.0001	123.5	240.0
317.4	0.21	21176	0.0016	0.0001	123.5	239.7
319.4	0.30	21607	0.0023	0.0001	123.4	240.1
321.3	0.13	7110	0.0030	0.0002	123.3	240.0
323.3	0.35	15597	0.0037	0.0001	123.3	238.9
325.2	0.17	5003	0.0055	0.0003	123.2	239.8
327.2	0.42	10108	0.0069	0.0002	123.1	239.3
329.1	0.26	4966	0.0088	0.0004	123.1	238.8
331.1	0.48	6804	0.0117	0.0004	123.0	238.8
333.0	0.48	5404	0.0150	0.0005	122.9	238.4
334.9	0.61	5300	0.0195	0.0006	122.9	238.4
336.9	0.83	5170	0.0272	0.0008	122.8	238.8
337.9	0.86	5409	0.0269	0.0008	122.8	237.5
338.8	1.26	6480	0.0330	0.0009	122.7	238.1
340.8	1.55	6603	0.0400	0.0010	122.7	237.4

2-methyl-5-nitrobenzoic acid (6-methyl-3-nitrobenzoic acid); Orifice – 0.2 mm

$\Delta_{cr}^{g}H_{m}^{o}$(298.15 K) = 122.9± 3.6 kJ·mol^{-1}

$\Delta_{cr}^{g}S_{m}^{o}$(298.15 K) = 233.7 ± 3.1 J·mol^{-1}·K^{-1}

$\Delta_{cr}^{g}G_{m}^{o}$(298.15 K) = 53.2 ± 0.1 kJ·mol^{-1}

$\ln(p_i\,/p_{ref}) = \frac{363.3}{R} - \frac{133014}{RT} - \frac{33.9}{R}\ln\frac{T}{298.15}$; p_{ref} = 1 Pa

359.1	0.24	37715	0.20	0.01	120.8	227.3
361.0	0.37	46261	0.25	0.01	120.8	227.2

363.0	0.51	49279	0.31	0.01	120.7	227.2
364.9	0.33	27083	0.38	0.01	120.6	226.8
366.9	0.45	30008	0.46	0.01	120.6	226.5
368.8	0.44	23598	0.58	0.01	120.5	226.6
370.8	0.53	22304	0.74	0.02	120.4	226.6
372.7	0.29	10242	0.86	0.02	120.4	226.1
374.7	0.34	10228	1.02	0.03	120.3	225.6
376.6	0.31	7278	1.31	0.04	120.2	225.9
378.5	0.37	7273	1.57	0.04	120.2	225.6
380.5	0.35	5548	1.96	0.05	120.1	225.6
382.4	0.39	5286	2.31	0.05	120.1	225.2

2-methyl-5-nitrobenzoic acid (6-methyl-3-nitrobenzoic acid); Orifice – 1.0 mm

$\Delta_{cr}^{g}H_{m}^{o}$(298.15 K) = 125.7± 3.2 kJ·mol^{-1}

$\Delta_{cr}^{g}S_{m}^{o}$(298.15 K) = 244.1 ± 2.7 J·mol^{-1}·K^{-1}

$\Delta_{cr}^{g}G_{m}^{o}$(298.15 K) = 53.8 ± 0.1 kJ·mol^{-1}

$$\ln(p_i/p_{ref}) = \frac{370.8}{R} - \frac{135768}{RT} - \frac{33.9}{R}\ln\frac{T}{298.15}; \; p_{ref} = 1\text{ Pa}$$

331.1	0.65	87600	0.006	0.0002	124.5	237.8
333.1	0.58	61756	0.008	0.0002	124.5	237.4
335.0	0.49	40040	0.010	0.0003	124.4	237.3
337.0	0.38	24844	0.012	0.0004	124.3	236.7
338.9	0.50	24840	0.016	0.0005	124.3	236.8
340.8	0.32	13000	0.020	0.0007	124.2	236.1
342.8	0.44	12860	0.028	0.0008	124.1	236.6
344.7	0.28	6780	0.034	0.0012	124.1	236.2
346.7	0.39	7070	0.045	0.0014	124.0	236.1
348.6	0.35	5094	0.057	0.0019	123.9	236.0
350.6	0.44	5070	0.072	0.0021	123.9	235.7
352.6	0.59	5290	0.092	0.0024	123.8	235.6
354.6	0.68	5020	0.113	0.0028	123.7	235.1
356.5	0.92	5280	0.145	0.0033	123.7	235.2
358.5	1.67	7990	0.174	0.0035	123.6	234.6

2-methyl-5-nitrobenzoic acid (6-methyl-3-nitrobenzoic acid); Orifice – 3.0 mm

$\Delta_{cr}^{g}H_{m}^{o}$(298.15 K) = 120.9± 6.4 kJ·mol^{-1}

$\Delta_{cr}^{g}S_{m}^{o}$(298.15 K) = 228.91 ± 5.3 J·mol^{-1}·K^{-1}

$\Delta_{cr}^{g}G_{m}^{o}$(298.15 K) = 52.7 ± 0.1 kJ·mol^{-1}

$$\ln(p_i/p_{ref}) = \frac{358.5}{R} - \frac{131013}{RT} - \frac{33.9}{R}\ln\frac{T}{298.15}; \; p_{ref} = 1\text{ Pa}$$

315.7	0.23	41946	0.00089	0.00004	120.3	227.0
317.2	0.37	56963	0.00106	0.00004	120.3	226.4
318.7	0.60	71023	0.00140	0.00005	120.2	226.9
320.2	0.41	40264	0.00169	0.00006	120.2	226.5

321.6	0.50	40460	0.00205	0.00007	120.1	226.2
323.1	0.33	22454	0.00242	0.00010	120.1	225.8
324.5	0.48	25470	0.00311	0.00011	120.0	226.1
326.0	0.23	10216	0.00381	0.00018	120.0	226.0
327.5	0.37	13227	0.00460	0.00017	119.9	225.7
328.9	0.25	7417	0.00574	0.00025	119.9	225.8
330.4	0.29	7501	0.00652	0.00027	119.8	225.1

2-methyl-4-nitrobenzoic acid; Orifice – 0.2 mm

$\Delta_{\mathrm{cr}}^{\mathrm{g}}H_{\mathrm{m}}^{\mathrm{o}}$(298.15 K) = 122.5± 4.6 kJ·mol^{-1}

$\Delta_{\mathrm{cr}}^{\mathrm{g}}S_{\mathrm{m}}^{\mathrm{o}}$(298.15 K) = 239.8 ± 3.8 J·mol^{-1}·K^{-1}

$\Delta_{\mathrm{cr}}^{\mathrm{g}}G_{\mathrm{m}}^{\mathrm{o}}$(298.15 K) = 51.0 ± 0.1 kJ·mol^{-1}

$\ln(p_i\,/p_{ref}) = \frac{369.4}{R} - \frac{132621}{RT} - \frac{33.9}{R}\ln\frac{T}{298.15}$; p_{ref} = 1 Pa

358.4	0.30	20420	0.45	0.01	120.5	233.8
360.4	0.39	22094	0.54	0.01	120.4	233.3
362.4	0.36	16172	0.68	0.02	120.3	233.2
364.3	0.43	16166	0.81	0.02	120.3	232.6
366.2	0.35	10241	1.05	0.03	120.2	232.9
368.2	0.42	10217	1.26	0.03	120.1	232.4
370.2	0.38	7190	1.64	0.04	120.1	232.7
372.2	0.45	7213	1.92	0.04	120.0	232.1
374.1	0.44	5476	2.51	0.06	119.9	232.5
376.4	0.50	5323	2.94	0.06	119.9	231.7
378.7	0.63	5371	3.66	0.07	119.8	231.4
380.7	0.78	5361	4.57	0.08	119.7	231.3
382.4	0.90	4894	5.78	0.09	119.7	231.7
384.7	1.12	5194	6.77	0.10	119.6	231.0

[a] Temperature in the cell measured with the standard uncertainty ($u(T)$ = 0.2 K).

[b] Mass loss of the sample measured by weighing with the standard uncertainty ($u(m)$ = 0.01 g).

[c] Duration of the effusion experiment.

[d] Vapour pressure at temperature T, calculated from the m according to the Knudsen formula.

[e] Standard uncertainties were calculated with $u(p_i$/Pa) = 0.005 +0.025(p_i/Pa).

[f] Calculated using the following equation: $\Delta_{\mathrm{cr}}^{\mathrm{g}}H_{\mathrm{m}}^{\mathrm{o}}(T) = -b + \Delta_{\mathrm{cr}}^{\mathrm{g}}C_{p,\mathrm{m}}^{\mathrm{o}} \times T$.

Uncertainty of the sublimation enthalpy $U(\Delta_{\mathrm{cr}}^{\mathrm{g}}H_{\mathrm{m}}^{\mathrm{o}})$ is the expanded uncertainty (0.95 level of confidence). Uncertainty includes uncertainties from the experimental conditions and the fitting equation, vapour pressures, and uncertainties from adjustment of sublimation enthalpy to the reference temperature T = 298.15 K.

[g] Calculated using the following equation: $\Delta_{\mathrm{cr}}^{\mathrm{g}}S_{\mathrm{m}}^{\mathrm{o}}(T) = \Delta_{\mathrm{cr}}^{\mathrm{g}}H_{\mathrm{m}}^{\mathrm{o}}/T + R \times \ln(p_{\mathrm{i}}/p^{\mathrm{o}})$ with p^{o} = 0.1 MPa.

Transpiration method

Absolute vapour pressures were measured using the transpiration method[39-40]. The main idea of this method is to saturate the gas stream flowing over the sample and to determine the amount of compound transferred by the gas within a given time.

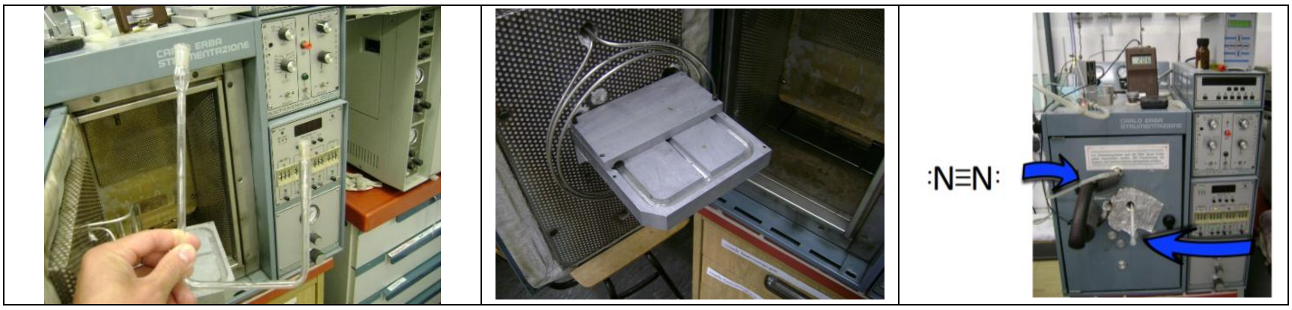

The high-temperature set-up for the transpiration measurements

Approximately 0.5 to 0.8 g of the sample is mixed with glass beads (diameter 1 mm) and placed in the thermostatted U-shaped saturator. The glass beads are needed to enlarge the contact area between gas and sample. A stream of nitrogen at a well-defined flow rate was passed through the saturator at a constant temperature (± 0.2 K) maintained by the modified oven of the commercial GC device and the transported material was collected in a cold trap. The amount of condensed substance was determined by weighing the glass trap using a balance with a resolution of 0.0001 g. The saturation vapour pressure p_i at each temperature T_i was calculated from the amount of condensate collected within a definite period of time:

$$p_i = m_i \cdot R \cdot T_a / V \cdot M_i \; ; \qquad V = (n_{N2} + n_i) \cdot R \cdot T_a / P_a \qquad \text{(S7)}$$

where V is the volume of the gas phase consisting of the n_{N2} moles of the carrier gas and n_i mole of gaseous compound under study (with the molar mass M_i) at the atmospheric pressure P_a and the ambient temperature T_a. The volume of the carrier gas V_{N2} was determined by the digital flow rate sensor from integration with a microcontroller. We used the Honeywell S&C - HAFBLF0200C2AX5 digital flow rate sensor with uncertainty at the level of 2.5 %. The flow rate of the nitrogen stream was also controlled by using a soap bubble flow meter (HP soap film flowmeter (model 0101-0113)) and optimized to reach the saturation equilibrium of the transporting gas at each temperature under study. The volume of the carrier gas V_{N2} was read from the digital flow sensor. The amount of the compound under investigation n_i in the carrier gas was estimated at each temperature using the ideal gas law.

Table S2. Absolute vapor pressures *p*, and standard thermodynamic functions of sublimation obtained by the transpiration method

T/ K[a]	m/ mg[b]	$V(N_2)$[c] / dm^3	Flow/ $dm^3 \cdot h^{-1}$	p/ Pa[d]	$u(p)$/ Pa[e]	$\Delta_{cr}^{g}H_{m}^{o}$/ $kJ \cdot mol^{-1}$	$\Delta_{cr}^{g}S_{m}^{o}$/ $J \cdot K^{-1} \cdot mol^{-1}$	

3-methyl-2-nitrobenzoic acid;

$\Delta_{cr}^{g}H_{m}^{o}$(298.15 K) = 125.7±1.1 $kJ \cdot mol^{-1}$;

$\Delta_{cr}^{g}S_{m}^{o}$(298.15 K) = 229.3±1.3 $J \cdot K^{-1} \cdot mol^{-1}$;

$\Delta_{cr}^{g}G_{m}^{o}$(298.15 K) = 57.3±0.1 $kJ \cdot mol^{-1}$

$$\ln(p/p_{ref}) = \frac{358.9}{R} - \frac{135781.6}{RT} - \frac{33.9}{R}\ln\frac{T}{298}\text{ ; } p_{ref} = 1\text{ Pa}$$

390.0	10.56	115.9	297.7	5.07	1.25	0.04	122.6	220.3
397.3	23.32	131.1	297.8	5.07	2.43	0.07	122.3	219.5
406.3	36.30	90.23	298.1	5.07	5.50	0.16	122.0	218.8
412.3	10.23	15.21	297.9	5.07	9.19	0.25	121.8	218.2
420.5	13.86	10.14	297.9	5.07	18.68	0.49	121.5	217.7
428.1	14.41	5.577	298.1	5.07	35.33	0.91	121.3	217.2
434.7	22.88	5.333	297.8	5.00	58.59	1.49	121.0	216.6
439.7	14.30	2.333	298.0	5.00	83.74	2.12	120.9	216.0
444.6	16.72	1.859	297.7	5.07	122.7	3.1	120.7	215.8

4-methyl-2-nitrobenzoic acid;

$\Delta_{cr}^{g}H_{m}^{o}$(298.15 K) = 120.9±1.6 $kJ \cdot mol^{-1}$;

$\Delta_{cr}^{g}S_{m}^{o}$(298.15 K) = 226.5±2.4 $J \cdot K^{-1} \cdot mol^{-1}$;

$\Delta_{cr}^{g}G_{m}^{o}$(298.15 K) = 53.4±0.1 $kJ \cdot mol^{-1}$

$$\ln(p/p_{ref}) = \frac{356.1}{R} - \frac{131009.8}{RT} - \frac{33.9}{R}\ln\frac{T}{298}\text{ ; } p_{ref} = 1\text{ Pa}$$

377.2	9.50	115.1	298.2	6.30	1.13	0.03	118.2	218.8
387.2	9.60	46.20	298.2	6.30	2.84	0.08	117.9	217.5
397.2	8.90	17.01	298.2	6.30	7.16	0.20	117.5	216.6
407.2	12.30	9.450	298.2	6.30	17.81	0.47	117.2	216.1
417.5	10.10	3.383	298.2	4.51	40.84	1.05	116.9	215.1
428.0	15.60	2.256	298.2	4.51	94.56	2.39	116.5	214.3

5-methyl-2-nitrobenzoic acid;

$\Delta_{cr}^{g}H_{m}^{o}$(298.15 K) = 123.3±1.6 $kJ \cdot mol^{-1}$;

$\Delta_{cr}^{g}S_{m}^{o}$(298.15 K) = 235.6±2.5 $J \cdot K^{-1} \cdot mol^{-1}$;

$\Delta_{cr}^{g}G_{m}^{o}$(298.15 K) = 53.0±0.1 $kJ \cdot mol^{-1}$

$$\ln(p/p_{ref}) = \frac{365.5}{R} - \frac{133386.4}{RT} - \frac{33.9}{R}\ln\frac{T}{298}\text{ ; } p_{ref} = 1\text{ Pa}$$

375.7	7.80	76.94	297.8	4.55	1.39	0.04	120.7	228.2
378.5	12.00	89.97	297.9	4.55	1.82	0.05	120.6	227.8
383.6	9.10	41.46	297.7	4.55	3.00	0.08	120.4	227.3
389.7	25.10	62.53	297.7	4.55	5.48	0.16	120.2	226.8

393.6	14.90	26.53	298.1	4.55	7.68	0.22	120.0	226.3
397.5	22.60	27.67	298.0	4.55	11.17	0.30	119.9	226.0
399.7	18.90	18.42	297.8	4.55	14.02	0.38	119.8	226.1
402.1	11.60	9.172	297.9	4.55	17.29	0.46	119.8	225.8

6-methyl-2-nitrobenzoic acid (crII);
$\Delta_{cr}^{g}H_{m}^{o}$(298.15 K) = 119.9±1.5 kJ·mol^{-1};
$\Delta_{cr}^{g}S_{m}^{o}$(298.15 K) = 227.6±1.8 J·K^{-1}·mol^{-1};
$\Delta_{cr}^{g}G_{m}^{o}$(298.15 K) = 52.1±0.1 kJ·mol^{-1}

$\ln\,(p/p_{\mathrm{ref}}) = \frac{357.2}{R} - \frac{130043.7}{RT} - \frac{33.9}{R}\ln\frac{T}{298}$; p_{ref} = 1 Pa

373.3	9.20	110.8	297.7	5.86	1.13	0.03	117.4	219.8
377.3	9.60	75.22	297.8	4.25	1.74	0.05	117.3	219.7
383.3	16.10	71.62	298.1	4.25	3.08	0.08	117.1	219.0
386.2	12.20	40.53	298.0	5.86	4.12	0.11	117.0	218.9
389.3	10.50	26.11	298.2	4.25	5.50	0.16	116.8	218.6
395.3	11.60	16.77	297.7	4.25	9.45	0.26	116.6	218.1
398.6	15.40	16.63	297.8	4.25	12.65	0.34	116.5	217.8
402.4	18.60	14.65	297.9	4.25	17.36	0.46	116.4	217.3

6-methyl-2-nitrobenzoic acid (crI);
$\Delta_{cr}^{g}H_{m}^{o}$(298.15 K) = 116.9±2.1 kJ·mol^{-1};
$\Delta_{cr}^{g}S_{m}^{o}$(298.15 K) = 219.3±2.2 J·K^{-1}·mol^{-1};
$\Delta_{cr}^{g}G_{m}^{o}$(298.15 K) = 51.5±0.1 kJ·mol^{-1}

$\ln\,(p/p_{\mathrm{ref}}) = \frac{349.0}{R} - \frac{127017.2}{RT} - \frac{33.9}{R}\ln\frac{T}{298}$; p_{ref} = 1 Pa

408.4	23.80	12.10	298.0	4.25	26.89	0.70	113.2	208.8
411.9	12.30	4.741	297.7	4.25	35.43	0.91	113.1	208.4
414.4	15.40	4.954	297.8	4.25	42.47	1.09	113.0	208.1
418.2	18.30	4.343	297.9	2.53	57.57	1.46	112.8	207.8
420.2	10.40	2.108	297.8	2.53	67.37	1.71	112.8	207.7
422.3	16.70	2.867	298.1	2.53	79.61	2.02	112.7	207.6
425.2	19.80	2.741	297.7	2.53	98.60	2.49	112.6	207.3
427.3	19.00	2.235	298.2	2.53	116.2	2.9	112.5	207.2

2-methyl-3-nitrobenzoic acid;
$\Delta_{cr}^{g}H_{m}^{o}$(298.15 K) = 120.6±1.4 kJ·mol^{-1};
$\Delta_{cr}^{g}S_{m}^{o}$(298.15 K) = 227.3±2.0 J·K^{-1}·mol^{-1};
$\Delta_{cr}^{g}G_{m}^{o}$(298.15 K) = 52.8±0.1 kJ·mol^{-1}

$\ln\,(p/p_{\mathrm{ref}}) = \frac{357.0}{R} - \frac{130657.8}{RT} - \frac{33.9}{R}\ln\frac{T}{298}$; p_{ref} = 1 Pa

384.0	15.70	80.83	297.9	5.13	2.66	0.07	117.6	218.8
400.3	10.60	12.13	297.9	5.13	11.94	0.32	117.1	217.4

404.1	14.20	11.96	297.7	5.13	16.21	0.43	117.0	216.9
409.2	9.00	4.871	298.1	5.13	25.27	0.66	116.8	216.5
415.2	9.70	3.247	297.8	5.13	40.81	1.05	116.6	215.9
419.2	11.40	2.820	297.7	5.13	55.20	1.41	116.4	215.4
422.9	13.60	2.341	298.0	4.53	79.38	2.01	116.3	215.7
425.7	23.70	3.250	298.1	5.13	99.65	2.52	116.2	215.6
425.7	15.30	2.224	298.0	5.13	94.00	2.37	116.2	215.1
428.0	12.00	1.369	298.0	5.13	119.8	3.0	116.2	215.5
429.1	29.50	3.333	297.8	5.13	120.8	3.1	116.1	214.7
432.1	15.90	1.359	298.0	4.53	159.7	4.0	116.0	215.0
432.6	20.50	1.711	298.0	5.13	163.6	4.1	116.0	214.8
434.2	37.60	2.905	298.0	5.13	176.7	4.4	115.9	214.3
438.1	17.60	0.982	298.0	4.53	244.6	6.1	115.8	214.3
443.3	27.40	1.057	298.0	4.53	353.2	8.8	115.6	213.9
448.3	29.80	0.831	298.0	4.53	488.2	12.2	115.5	213.3

4-methyl-3-nitrobenzoic acid;
$\Delta_{cr}^{g}H_{m}^{o}$(298.15 K) = 121.2±1.6 kJ·mol^{-1};
$\Delta_{cr}^{g}S_{m}^{o}$(298.15 K) = 224.0±2.3 J·K^{-1}·mol^{-1};
$\Delta_{cr}^{g}G_{m}^{o}$(298.15 K) = 54.4±0.1 kJ·mol^{-1}

$$\ln\,(p/p_{\mathrm{ref}}) = \frac{353.6}{R} - \frac{131261.2}{RT} - \frac{33.9}{R}\ln\frac{T}{298}\ ;\ p_{\mathrm{ref}} = 1\ \mathrm{Pa}$$

389.4	14.00	78.30	298.0	4.14	2.45	0.07	118.1	214.9
398.5	17.20	43.05	298.0	4.14	5.46	0.16	117.8	213.9
408.4	18.10	18.63	298.0	4.14	13.29	0.36	117.4	213.3
418.5	14.70	6.277	298.0	4.14	32.01	0.83	117.1	212.8
424.3	8.10	2.276	298.0	4.14	48.64	1.24	116.9	212.1
428.8	12.10	2.414	298.0	4.14	68.49	1.74	116.7	211.7
432.6	13.60	2.070	298.0	4.14	89.79	2.27	116.6	211.2
438.8	17.20	1.656	298.0	4.14	141.9	3.6	116.4	210.7

6-methyl-3-nitrobenzoic acid;
$\Delta_{cr}^{g}H_{m}^{o}$(298.15 K) = 118.4±1.6 kJ·mol^{-1};
$\Delta_{cr}^{g}S_{m}^{o}$(298.15 K) = 220.5±1.9 J·K^{-1}·mol^{-1};
$\Delta_{cr}^{g}G_{m}^{o}$(298.15 K) = 52.7±0.1 kJ·mol^{-1}

$$\ln\,(p/p_{\mathrm{ref}}) = \frac{350.1}{R} - \frac{128547.8}{RT} - \frac{33.9}{R}\ln\frac{T}{298}\ ;\ p_{\mathrm{ref}} = 1\ \mathrm{Pa}$$

403.7	12.10	12.50	297.7	4.36	13.22	0.36	114.9	210.3
409.7	11.70	7.486	297.8	4.36	21.35	0.56	114.7	209.6
415.8	12.70	4.870	298.2	4.36	35.68	0.92	114.5	209.3
420.9	9.30	2.399	298.1	4.36	53.01	1.35	114.3	208.8
426.8	11.20	1.817	297.9	4.36	84.19	2.13	114.1	208.5
432.7	20.10	2.108	298.0	4.36	130.2	3.3	113.9	208.0
438.9	21.20	1.454	297.8	4.36	198.9	5.0	113.7	207.3

2-methyl-4-nitrobenzoic acid;

$\Delta_{cr}^{g}H_{m}^{o}$(298.15 K) = 112.5±1.5 kJ·mol^{-1};
$\Delta_{cr}^{g}S_{m}^{o}$(298.15 K) = 212.0±2.2 J·K^{-1}·mol^{-1};
$\Delta_{cr}^{g}G_{m}^{o}$(298.15 K) = 49.3±0.1 kJ·mol^{-1}

$\ln(p/p_{ref}) = \frac{341.6}{R} - \frac{122600.3}{RT} - \frac{33.9}{R}\ln\frac{T}{298}$; p_{ref} = 1 Pa

376.4	14.80	75.52	297.9	4.72	2.68	0.07	109.8	204.3
382.4	30.90	94.20	297.9	4.89	4.48	0.12	109.6	203.5
389.6	13.30	21.33	298.1	4.89	8.53	0.24	109.4	202.9
395.6	14.70	14.25	298.2	4.89	14.12	0.38	109.2	202.3
400.6	11.60	7.572	298.0	4.89	20.95	0.55	109.0	201.7
405.5	22.40	9.676	297.8	4.72	31.63	0.82	108.9	201.5
408.7	11.00	3.619	297.7	4.72	41.51	1.06	108.7	201.4
412.1	17.40	4.397	297.8	4.89	54.06	1.38	108.6	201.1
416.8	20.80	3.619	297.8	4.72	78.49	1.99	108.5	200.8

3-methyl-4-nitrobenzoic acid;
$\Delta_{cr}^{g}H_{m}^{o}$(298.15 K) = 125.0±1.3 kJ·mol^{-1};
$\Delta_{cr}^{g}S_{m}^{o}$(298.15 K) = 231.9±1.5 J·K^{-1}·mol^{-1};
$\Delta_{cr}^{g}G_{m}^{o}$(298.15 K) = 55.9±0.1 kJ·mol^{-1}

$\ln(p/p_{ref}) = \frac{361.5}{R} - \frac{135138.7}{RT} - \frac{33.9}{R}\ln\frac{T}{298}$; p_{ref} = 1 Pa

398.6	14.10	42.24	298.2	4.83	4.57	0.12	121.6	222.0
403.5	9.80	18.91	298.2	4.83	7.09	0.20	121.5	221.6
410.7	30.50	31.78	298.2	4.83	13.13	0.35	121.2	220.9
415.7	19.90	13.44	298.2	4.83	20.26	0.53	121.0	220.5
419.5	11.80	5.633	298.2	4.83	28.66	0.74	120.9	220.4
422.7	22.90	8.529	298.2	4.83	36.73	0.94	120.8	220.0
425.8	15.70	4.506	298.2	4.83	47.66	1.22	120.7	219.9
430.6	33.20	6.598	298.2	4.83	68.81	1.75	120.5	219.4
437.7	11.40	1.328	298.2	4.83	117.4	3.0	120.3	218.8

[a] Saturation temperature measured with the standard uncertainty ($u(T)$ = 0.1 K).
[b] Mass of transferred sample condensed at T = 273 K.
[c] Volume of nitrogen ($u(V)$ = 0.005 dm^3) used to transfer m ($u(m)$ = 0.0001 g) of the sample. Uncertainties are given as standard uncertainties.
[d] Vapor pressure at temperature T, calculated from the m and the residual vapor pressure at the condensation temperature calculated by an iteration procedure.
[e] Standard uncertainties were calculated with $u(p_i/\text{Pa})$ = 0.005 +0.025(p_i/Pa) for pressures below 5 Pa, and $u(p_i/\text{Pa})$ = 0.025 + 0.025(p_i/Pa) for pressures from 5 to 3000 Pa. The standard uncertainties for T, V, p, m, are standard uncertainties with 0.683 confidence levels. Uncertainty of the sublimation enthalpy $U(\Delta_{cr}^{g}H_{m}^{o})$ is the expanded uncertainty (0.95 level of confidence) calculated according to procedure described elsewhere. Uncertainties include uncertainties from the experimental conditions and the fitting equation, vapor pressures, and uncertainties from adjustment of vaporisation enthalpies to the reference temperature T = 298.15 K.

Differential scanning calorimetry: temperatures and enthalpies of fusion

DSC Mettler Toledo DSC 822e: The thermal behavior of crystalline samples of MNBAs including melting temperature T_{fus}, and the standard molar enthalpy of fusion, $\Delta_{cr}^{l}H_{m}^{o}(T_{fus})$, was studied with a commercial DSC Mettler Toledo DSC 822e coupled with Huber TC100MT cooler. The sample was placed in the standard non-pinned aluminium pan of 40 µl volume. The pan and sample were weighed with the Sartorius MSE3.6P-000-DM microbalance with the standard uncertainty of $5\cdot10^{-6}$ g. The calibration of the DSC was checked with melting behaviour of reference indium sample. The twice standard deviation of the enthalpy of fusion in the test measurements for reference compound was ± 0.3 $kJ\cdot mol^{-1}$ and ± 0.3 K for the melting temperature. Uncertainties of the enthalpy of fusion values are expressed as expanded uncertainties (at a level of confidence of 0.95, k=2). They include uncertainties from fusion experiments and calibration. Details are reported elsewhere[41].
In the first DSC run the sample was heated with a rate 10 $K\cdot min^{-1}$ to 378 K (~ 20 K above melting temperature provided by supplier) and then cooled down to 298 K also with the rate10 $K\cdot min^{-1}$. Such procedure provided sufficient contact between the sample and the bottom of pan. The DSC experiments were repeated three times with the average results shown in Table S3. The uncertainties of the melting temperature and the enthalpy of fusion are expressed as an expanded uncertainties with a confidence level of 95 % (k ≈ 2). The melting temperature was evaluated as the onset temperature of the melting peak adjusted to the zero-heating rate.

As a rule, thermochemical calculations are commonly performed at the reference temperature T = 298.15 K. The adjustment of $\Delta_{cr}^{l}H_{m}^{o}(T_{fus})$ was performed with help of the equation [13]:

$$\Delta_{cr}^{l}H_{m}^{o}(298.15\text{ K})/(\text{J}\cdot\text{mol}^{-1}) = \Delta_{cr}^{l}H_{m}^{o}(T_{fus}/\text{K}) - (\Delta_{cr}^{g}C_{p,m}^{o}-\Delta_{l}^{g}C_{p,m}^{o})\times[(T_{fus}/\text{K}) - 298.15\text{ K}] \qquad \text{(S8)}$$

where $\Delta_{cr}^{g}C_{p,m}^{o}$ and $\Delta_{l}^{g}C_{p,m}^{o}$ were calculated according to the Acree and Chickos.[13] With this adjustment, the molar enthalpies of fusion, $\Delta_{cr}^{l}H_{m}^{o}$(298.15 K) were calculated. Uncertainties in the temperature adjustment of fusion enthalpy from T_{fus} to the reference temperature were estimated to account with 30 % to the total adjustment.[13]

Perkin-Elmer Pyris Diamond DSC. The thermal behavior of MNBAs including phase transition and enthalpy of fusion was studied with a computer controlled differential scanning calorimeter Perkin-Elmer Pyris Diamond DSC. The sample was hermetically sealed in 50 µL pan supplied by Perkin Elmer. For all measurements, an empty pan was used as reference. The fusion temperature and the enthalpy were determined as the peak onset temperature and by using a straight baseline for integration, respectively. The temperature and heat flow rate scale of the DSC were calibrated by measuring high-purity indium (T_0 =429.8 K and ΔH_{ref} = 28.4 $J\cdot g^{-1}$). The thermal behaviour of the specimen was investigated at heating rate of 10 $K\cdot min^{-1}$. The uncertainty for temperature is ±0.5 K and for enthalpy of fusion ±1 $J\cdot g^{-1}$. The DSC measurements were repeated twice and values agreed within the experimental uncertainties ± 0.2 $kJ\cdot mol^{-1}$ for the enthalpy of fusion and ± 0.5 K for the melting temperature. Uncertainties in the temperature adjustment of fusion enthalpies from T_{fus} to the reference temperature were assumed to amount to 30% of the total adjustment [13] . The average DSC results shown in Table S3.

Table S3. Compilation of enthalpies of fusion of methyl-nitrobenzoic acids (in kJ·mol^{-1}) and *W*alden´s *C*onstants (in J·K^{-1}·mol^{-1}.)

Compound	T_{fus}, K	$\Delta_{cr}^{l}H_{m}^{o}$ [a] at T_{fus}		WC [b]
3-methyl-2-nitrobezoic acid	496.7	35.7±0.3		71.9±0.4
4-methyl-2-nitrobezoic acid	428.0	24.7±0.3		57.7±0.4
5-methyl-2-nitrobezoic acid	408.9	21.4±0.3		52.3±0.4
6-methyl-2-nitrobezoic acid	428.3	26.7±0.5		62.3±0.6
2-methyl-3-nitrobezoic acid	456.8	31.5±0.2		69.4±0.3
4-methyl-3-nitrobezoic acid	464.2	28.0±0.2		60.3±0.3
6-methyl-3-nitrobezoic acid	451.4	27.2±0.2		60.7±0.3
2-methyl-4-nitrobezoic acid	428.7	21.2±0.4		49.5±0.5
3-methyl-4-nitrobezoic acid	490.0	33.8±0.3		69.0±0.4
			average	**61.5±2.6** [c]

[a] The evaluated experimental enthalpies of fusion $\Delta_{cr}^{l}H_{m}^{o}$ at T_{fus} from Table 2 and are presented in kJ·mol^{-1}.

[b] The *W*alden´s *C*onstant[16]: $WC = \frac{\Delta_{cr}^{l}H_{m}^{o}}{T_{fus}} = \Delta_{cr}^{l}S_{m}^{o}$ in J·K^{-1}·mol^{-1}.

[c] The weighted average value (uncertainties were taken as the weighing factor).

Non-Isothermal Thermogravimetry

A Stanton-Redcroft 625 simultaneous TG/DSC apparatus connected to a personal computer was used. The system was calibrated using several high-purity standards, including tin ref and indium ref, whose melting temperature and fusion enthalpies are well known. The uncertainties of temperature and mass measurements are the following: μ(T) = ± 0.5 K; μ(m) = ± 0.0005 mg. Increasing temperature experiments were carried, under a stream of argon, from room temperature to about 600 K at 5 K·min^{-1}. The uncertainty in the temperature measurements was estimated to be ± 0.01 K for all the experiments. An open aluminum crucible, with a cross-sectional area of 2.0·10^{-5} m^2, containing the sample and an empty one of equivalent mass as the reference were used. Samples with sizes of about (4 to 6) mg were placed in the crucible. The surface area of the molten compounds was considered equal to the area of the crucible bottom. The simultaneous TG/DSC system was flushed with a pure gas stream to remove the vapor during the vaporization of the sample.

The vaporization enthalpy of a compound can be determined by the temperature dependence of its vapor pressure. The vapor pressure p of a compound at a temperature T is related to its mass-loss rate $\Delta m/\Delta t$ by the Langmuir equation[42]. The temperature dependence of the rate of mass loss is derived both by the first derivative of the NI-TG data at 5 K·min^{-1} and further derived as described in the experimental section of the article. Adjustment to the reference temperature is done in accordance with the method proposed by Chickos *et al*.[38] using $C_{p,m}^{o}$(l) = 292.2 J·K^{-1}·mol^{-1}. From the slope of the mass vs time curve, a *dm/dt* relation can be obtained which will then be used in the adapted Clausius-Clapeyron equation to determine their respective molar enthalpies of vaporization:

$$\ln\left(\frac{dm}{dt}\sqrt{T}\right) = -\frac{\Delta_{l}^{g}H_{m}}{RT} + A \qquad \text{(S9)}$$

Table S4. Summary of the NITG experiments

Compounds	N[a]	T_{range} (K)	T_{av} (K)	m[b]	R^2	$\Delta_l^g H_m(T_{av})$[c]	$\Delta_l^g H_m(298.15\ K)$[d]
2M3NBA	240	[471.2-491.2]	481.2	-9989±42	0.9958	83.0 ± 0.7	99.2 ± 3.2
2M5NBA	238	[468.2-488.3]	478.3	-10084±10	0.9998	83.8 ± 0.2	99.8 ± 3.2
2M6NBA	241	[450.4-470.8]	460.7	-10340±8	0.9999	86.0 ± 0.1	100.4 ± 2.9
5M2NBA	237	[443.2-463.1]	453.2	-10784±10	0.9998	89.7 ± 0.2	103.4 ± 2.7

[a] N is the total number of NITG points used to develop the fitting equation.
[b] The slope the linear regression of *ln* Q vs 1/*T* plot. The uncertainties are standard deviations with a confidence level of 0.66.
[c] The standard molar enthalpy of vaporisation at the average temperature of the NITG experiment. The uncertainties are twice the standard deviations, with a confidence level of 0.95.
[d] The standard molar enthalpy of vaporisation at the reference temperature *T* = 298.15 K. Uncertainties of the vaporisation enthalpies $U(\Delta_l^g H_m^o)$ are the expanded uncertainties (0.95 level of confidence). They include uncertainties from the fitting equation and uncertainties from temperature adjustment to *T* = 298.15 K, which are estimated to account with 20 % to the total adjustment

Table S5. The enthalpies of sublimation/vaporisation, $\Delta_{cr,l}^{g} H_m^o$, enthalpies of fusion, $\Delta_{cr}^{l} H_m^o$, and enthalpies of formation, $\Delta_f H_m^o$(cr/liq/gas), of the auxiliary reference compounds at *T* = 298.15 K (*p*° = 0.1 MPa) in kJ·mol^{-1}

Compounds	$\Delta_{cr,l}^{g} H_m^o$	$\Delta_f H_m^o$(cr or liq)	$\Delta_f H_m^o$(g)
Benzene (liq)[43]	33.9±0.1		82.9±0.9
Toluene (liq)[43]	38.1±0.2		50.1±1.1
Nitrobenzene(liq)[44]	55.0±0.1		65.6±1.6
Benzoic acid (liq)	75.8±0.8 [41]	-371.4±1.3 [41]	-294.1±2.2 [45]
2-Nitro-toluene (liq) [8]	58.8±0.4	-21.7±0.9	37.1±1.0
3-Nitro-toluene (liq) [8]	59.4±0.4	-30.4±1.5	29.0±1.5
4-Nitro-toluene (liq) [8]	60.4±0.6		29.9±1.1
2-Nitrobenzoic acid (cr)	96.9±2.0 [Table S8]	-399.0±0.6 [5]	-280.3±0.8
3-Nitrobenzoic acid (cr)	97.0±2.0 [Table S8]	-414.0±0.4 [5]	-304.0±0.6
4-Nitrobenzoic acid (cr)	94.2±3.6 [Table S8]	-427.2±0.8 [5]	-307.5±1.0
2-Methylbenzoic acid (cr) [7]	79.8±0.8		-320.3±0.9
3-Methylbenzoic acid (cr) [7]	83.3±0.8		-328.9±0.9
4-Methylbenzoic acid (cr) [7]	81.9±1.0		-328.5±2.6

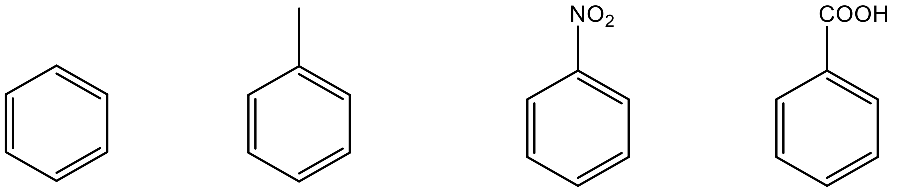

33.9 ± 0.1	38.1 ± 0.2<br>$38.1 - 33.9 = 4.2$<br>$(H \rightarrow CH_3) = 4.2$	55.0 ± 0.1<br>$55.0 - 33.9 = 21.1$<br>$(H \rightarrow NO_2) = 21.1$	75.8 ± 0.8

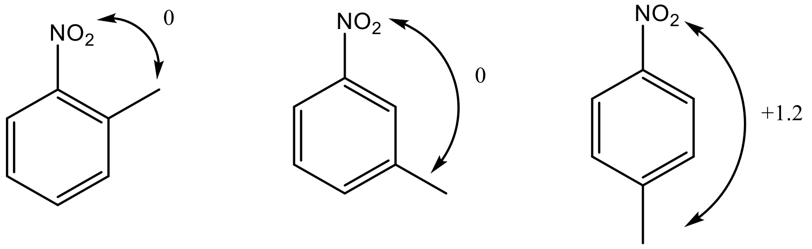

58.8 ± 0.4 (exp.)	59.4 ± 0.4 (exp.)	60.4 ± 0.6 (exp.)
Framework:<br>33.3 + 21.1 + 4.2 = 59.2<br>ortho (CH_3-NO_2)<br>58.8 - 59.2 = (-0.4) ≡ 0	Framework:<br>33.3 + 21.1 + 4.2 = 59.2<br>meta (CH_3-NO_2)<br>59.4 - 59.2 = (-0.2) ≡ 0	Framework:<br>33.3 + 21.1 + 4.2 = 59.2<br>para (CH_3-NO_2)<br>60.4 - 59.2 = (+1.2)

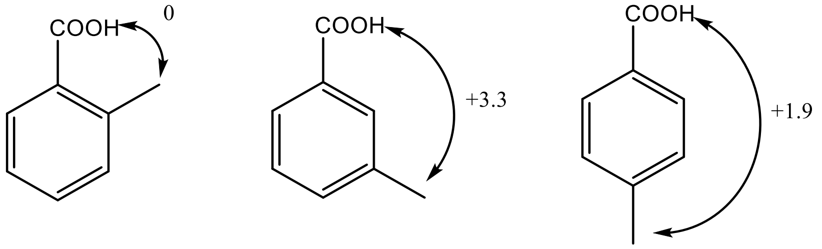

79.8 ± 0.8 (exp.)	83.3 ± 0.8 (exp.)	81.9 ± 1.0 (exp.)
Framework:<br>75.8 + 4.2 = 80.0<br>ortho (CH_3-COOH)<br>79.8 - 80.0 = (-0.2) ≡ 0	Framework:<br>75.8 + 4.2 = 80.0<br>meta (CH_3-COOH)<br>83.3 - 80.0 = (+3.3)	Framework:<br>75.8 + 4.2 = 80.0<br>para (CH_3-COOH)<br>81.9 - 80.0 = (+1.9)

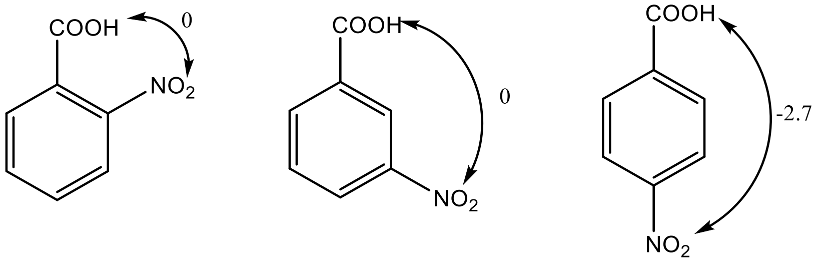

96.9 ± 2.0 (exp.)	97.0 ± 2.0 (exp.)	94.2 ± 3.6 (exp.)
Framework:<br>75.8 + 21.1 = 96.9<br>ortho (NO_2-COOH)<br>96.9 - 96.9 = 0	Framework:<br>75.8 + 21.1 = 96.9<br>meta (NO_2-COOH)<br>97.0 - 96.9 = (0.1) ≡ 0	Framework:<br>75.8 + 21.1 = 96.9<br>para (CH_3-COOH)<br>94.2 - 96.9 = (-2.7)

Fig. S2 Development of the increments and pairwise interactions of substituents on the benzene ring required for estimation of enthalpies of vaporisation, $\Delta_l^g H_m^o$(298 K), of MNBAs using benzoic acid as the "centerpiece". All data are from Table S5 (in kJ·mol^{-1})

Combustion calorimetry: enthalpy of formation determination

The standard molar energies of combustion were measured with a self-made high-precision isoperibolic calorimeter with a static bomb and a stirred water bath. About 0.3 g of the solid sample was pressed into a pellet, weighed with a microbalance with a resolution of 10^{-6} g, and burned in oxygen at a pressure of 3.04 MPa. In a preliminary series of combustion experiments, a considerable amount of soot was sometimes observed on the walls of the Pt crucible. To achieve complete combustion, a certain amount of polyethylene cuttings was used as an auxiliary compound. The pellet was placed in the crucible, surrounded with polyethylene cuts and ignited. The clean experiments with and without polyethylene showed results that agreed within the experimental uncertainty. The bomb was not purged with oxygen beforehand. Test experiments with and without purging with O_2 showed results that were also consistent within the experimental uncertainty. The detailed procedure has already been described[46] [47]. The combustion products were examined for carbon monoxide (Dräger tube) and unburned carbon, but neither was detected. The energy equivalents of the calorimeter ε_{calor} = (14799.65±1.15) J·K^{-1} for 2-methyl-4-nitrobenzoic acid, 6-methyl-3-nitrobenzoic acid, and 5-methyl-2-nitrobenzoic acid and ε_{calor} = (14800.86±1.11) J·K^{-1} for other methyl-nitrobenzoic acids were determined with a standard reference sample of benzoic acid (sample SRM 39*j*, NIST). Correction for nitric acid formation was based on titration with 0.1 mol·dm^{-3} NaOH (aq). Conventional procedures[48] were used for reduction of the data to standard conditions. Auxiliary data required for the reduction are collected in Table S6. The total uncertainties of $\Delta_c H_m^o$ and $\Delta_f H_m^o$-values calculated according to the guidelines presented by Hubbard *et al.*[48] and Olofsson[49]. The uncertainties of combustion energies, $\Delta_c u^o$(cr), are expressed as standard deviation of the mean. According to the thermochemical practice, the uncertainties assigned to the $\Delta_f H_m^o$(cr)-values are twice the overall standard deviations and include the uncertainties of the calibration, the combustion energies of the auxiliary materials, and the uncertainties of the enthalpies of formation of the reaction products $H_2O_{(liq)}$ (-285.830 ± 0.040 kJ·mol^{-1}) and $CO_{2(gas)}$ (-393.51 ± 0.13 kJ·mol^{-1}) as assigned by CODATA.[49]

Table S6 Auxiliary quantities: formula, density ρ(293 K), massic heat capacity c_p(298.15 K), and expansion coefficients $(\delta V/\delta T)_p$ of the materials used in the present study [a]

Materials	Formula	ρ (293 K)	c_p(298.15 K)[b]	$(\delta V/\delta T)_p$ [c]
		g·cm^{-3}	J·K^{-1} g^{-1}	10^{-3}·cm^3·K^{-1}·g^{-1}
3-methyl-2-nitrobezoic acid	$C_8H_7NO_4$	1.48 [d]	1.22	0.1
4-methyl-2-nitrobezoic acid	$C_8H_7NO_4$	1.477[50]	1.22	0.1
5-methyl-2-nitrobezoic acid	$C_8H_7NO_4$	1.48 [d]	1.22	0.1
6-methyl-2-nitrobezoic acid	$C_8H_7NO_4$	1.48 [d]	1.22	0.1
2-methyl-3-nitrobezoic acid	$C_8H_7NO_4$	1.48 [d]	1.22	0.1
4-methyl-3-nitrobezoic acid	$C_8H_7NO_4$	1.479 [51]	1.22	0.1
6-methyl-3-nitrobezoic acid	$C_8H_7NO_4$	1.494 [50]	1.22	0.1
2-methyl-4-nitrobezoic acid	$C_8H_7NO_4$	1.48 [d]	1.22	0.1
3-methyl-4-nitrobezoic acid	$C_8H_7NO_4$	1.48	1.22	0.1
polyethylene	$CH_{1.93}$	0.92	2.53	0.1
cotton	$CH_{1.774}O_{0.887}$	1.50	1.67	0.1

[a] Data for the densities, ρ(293 K), and the specific heat capacities, c_p(298.15 K), of auxiliary materials are from our previous work[52]. The specific energy of combustion $\Delta_c u^o$(cotton) = -16945.2 J·g^{-1}; $u(\Delta_c u^o)$ = 4.2 J·g^{-1}. The specific energy of combustion $\Delta_c u^o$(polyethylene) = -46357.3 J·g^{-1}; $u(\Delta_c u^o)$ = 3.1 J·g^{-1} from ten experiments according to the conventional procedure[48]. The standard uncertainties are reported for the specific energies of combustion.

[b] Calculated from the molar heat capacity at 298.15 K (see text).

[c] Estimated.

[d] Calculated as the mean value from the densities reported in references [50-51, 53]

Table S7. The typical combustion experiments at T = 298.15 K ($p°$ = 0.1 MPa) of the methyl-nitrobenzoic acids[a]

	3Me2NO2BA	4Me2NO2BA	5Me2NO2BA	6Me2NO2BA	2Me3NO2BA
m (substance) /g	0.422108	0.473867	0.508019	0.318537	0.197790
m'(cotton) /g	0.001117	0.001185	0.001166	0.001082	0.001050
m''(polyethylene) /g	0.331911	0.336656	0.355506	0.335241	0.462755
ΔT_c /K [b]	1.62728	1.71359	1.82096	1.49487	1.72577
$(\varepsilon_{calor})\cdot(-\Delta T_c)$ /J	-24085.1	-25362.6	-26949.6	-22125.3	-25542.9
$(\varepsilon_{cont})\cdot(-\Delta T_c)$ /J	-26.09	-27.89	-29.75	-23.62	-28.07
ΔU_{decomp} HNO_3 /J	48.68	51.36	53.16	44.50	46.59
ΔU_{corr} /J	12.2	13.30	14.30	10.20	9.99
$-m'\cdot\Delta_c u'$ /J	18.93	20.08	19.76	18.33	17.79
$-m''\cdot\Delta_c u''$ /J	15386.5	15606.5	16480.3	15540.9	21452.1
$\Delta_c u°$(cr) /(J·g^{-1})	-20480.5	-20468.5	-20494.9	-20516.0	-20448.7

Table S7 (continued). The typical combustion experiments at T = 298.15 K ($p°$ = 0.1 MPa) of the methyl-nitrobenzoic acids[a]

	4Me3NO2BA	6Me3NO2BA	2Me4NO2BA	3Me4NO2BA
m (substance) /g	0.403395	0.209394	0.220919	0.673841
m'(cotton) /g	0.001028	0.001189	0.001005	0.001076
m''(polyethylene) /g	0.328371	0.177966	0.442911	0.217433
ΔT_c /K [b]	1.58748	1.78957	1.69519	1.61299
$(\varepsilon_{calor})\cdot(-\Delta T_c)$ /J	-23496.1	-26485.0	-25088.3	-23873.6
$(\varepsilon_{cont})\cdot(-\Delta T_c)$ /J	-25.7	-29.32	-27.60	-25.84
ΔU_{decomp} HNO_3 /J	47.18	49.87	43.00	52.56
ΔU_{corr} /J	11.82	10.46	10.15	15.24
$-m'\cdot\Delta_c u'$ /J	17.42	20.15	17.03	18.23
$-m''\cdot\Delta_c u''$ /J	15222.4	22157.2	20532.2	10079.61
$\Delta_c u°$(cr) /(J·g^{-1})	-20384.6	-20423.9	-20430.5	-20381.4

[a] Results are referenced to T = 298.15 K ($p°$ = 0.1 MPa). The definition of the symbols assigned according to ref. [48] as follows: m(substance), m'(cotton) and m''(polyethylene) are, respectively, the mass of compound burnt, the mass of fuse (cotton) and auxiliary polyethylene used in each experiment, masses were corrected for buoyancy; V(bomb) = 0.33 dm^3 is the internal volume of the calorimetric bomb; p^i(gas) = 3.04 MPa is the initial oxygen pressure in the bomb; $m^i(H_2O)$ = 1.00 g is the mass of water added to the bomb for dissolution of combustion gases; ε_{calor} = (14832.2 ± 0.9) J·K^{-1}, uncertainty for the energy equivalent is expressed as the standard uncertainty; $\Delta T_c = T^f - T^i - \Delta T_{corr}$ is the corrected temperature rise from initial temperature T^i to final temperature T^f, with the correction ΔT_{corr} for heat exchange during the experiment; ε_{cont} is the energy equivalents of the bomb contents in their initial ε^i_{cont} and final states ε^f_{cont}, the contribution for the bomb content is calculated with $(\varepsilon_{cont})\cdot(-\Delta T_c) = (\varepsilon^i_{cont})\cdot(T^i - 298.15) + (\varepsilon^f_{cont})\cdot(298.15 - T^f + \Delta T_{corr.})$; ΔU_{decomp} HNO_3 is the energy correction for the nitric acid formation. ΔU_{corr} is the correction to standard states. Auxiliary data are given in Table S6.

Table S8. Phase transitions thermodynamics of methyl-substituted nitrobenzoic acids (in kJ·mol^{-1})[a]

Compounds	T_{fus}, K	$\Delta_{cr}^{l}H_{m}^{o}$ [b] at T_{fus}	$\Delta_{cr}^{l}H_{m}^{o}$ [c]	$\Delta_{cr}^{g}H_{m}^{o}$ [d]	$\Delta_{l}^{g}H_{m}^{o}$ [e]
			298.15 K		
1	2	3	4	5	6
2-nitrobezoic acid	419.0	28.0±0.5 [54]	21.8±1.9	118.7±0.5 [8]	96.9±2.0
3-nitrobezoic acid	414.3	19.3±0.5 [54]	13.0±2.0	110.0±0.4 [8]	97.0±2.0
4-nitrobezoic acid	512.4	36.9±0.5 [54]	25.5±3.5	119.7±0.6 [8]	94.2±3.6

[a] Uncertainties are presented as expanded uncertainties (0.95 level of confidence with k=2).

[b] The experimental enthalpies of fusion $\Delta_{cr}^{l}H_{m}^{o}$ measured at T_{fus} were adjusted to T = 298.15 K with help of the equation [13]:

$\Delta_{cr}^{l}H_{m}^{o}$(298.15 K)/(J·mol^{-1}) = $\Delta_{cr}^{l}H_{m}^{o}$(T_{fus}/K) – ($\Delta_{cr}^{g}C_{p,m}^{o}$-$\Delta_{l}^{g}C_{p,m}^{o}$)×[($T_{fus}$/K) – 298.15 K]

where $\Delta_{cr}^{g}C_{p,m}^{o}$ and $\Delta_{l}^{g}C_{p,m}^{o}$ were estimated according to the empirical procedures developed by Chickos *et al.* [13]. Uncertainties in the temperature adjustment of fusion enthalpies from T_{fus} to the reference temperature are estimated to account with 30 % to the total adjustment [13].

[c] The experimental enthalpies of fusion $\Delta_{cr}^{l}H_{m}^{o}$ at the reference temperature T = 298.15 K.

[d] Experimental values from [8]

[e] Calculated as the difference of column 5 and 4 in this table.

.

Table S9. Compilation of data on molar heat capacities $C_{p,m}^{o}$(g, cr or liq) and heat capacity differences $\Delta_{cr,l}^{g}C_{p,m}^{o}$ at T = 298.15 K (in J·K^{-1}·mol^{-1})

Compounds	$C_{p,m}^{o}$(cr) [a]	$-\Delta_{cr}^{g}C_{p,m}^{o}$ [b]	$C_{p,m}^{o}$(l) [a]	$-\Delta_{l}^{g}C_{p,m}^{o}$ [b]
2-nitrobezoic acid	191.6 [54]	29.5	271.3	81.1
3-nitrobezoic acid	173.2 [54]	26.7	271.3	81.1
4-nitrobezoic acid	180.3[54]	27.8	271.3	81.1

[a] The experimental values or calculated by the group-contribution procedure developed by Chickos *et al.*[38]

[b] Calculated according to the empirical procedure by Acree and Chickos [13]

Table S10. Structures of the stable conformers of methyl-benzoic acids, nitrobenzene, methyl-nitrobenzenes and nitrobenzoic acids

Front view	Top view
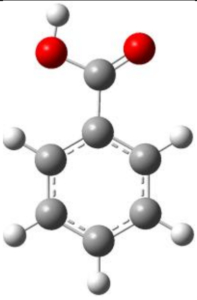	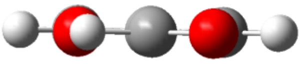
Benzoic acid CAS 65-85-0	
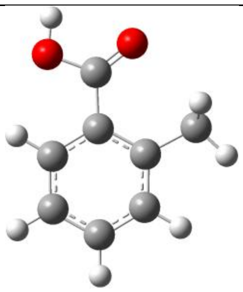	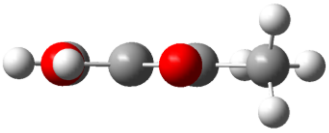
Relative energy, **0.0** kJ·mol^{-1}	
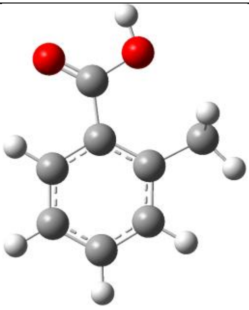	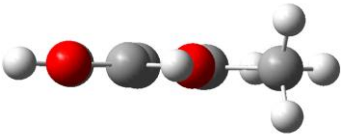
Relative energy, **4.9** kJ·mol^{-1}	
2-methylbenzoic acid CAS 118-90-1	
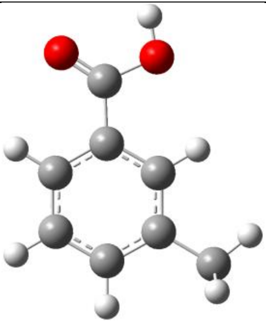	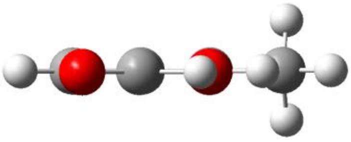
Relative energy, **0.0** kJ·mol^{-1}	
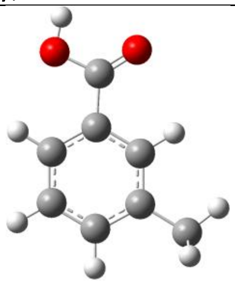	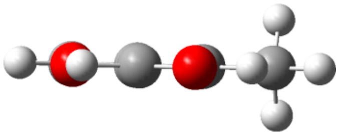

Relative energy, **0.3** kJ·mol^{-1}

3-methylbenzoic acid CAS 99-04-7

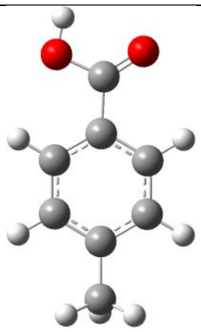

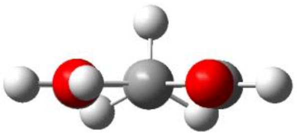

4-methylbenzoic acid CAS 99-94-5

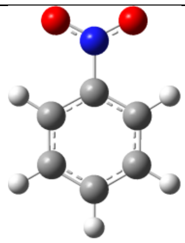

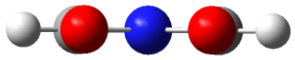

Nitrobenzene CAS 98-95-3

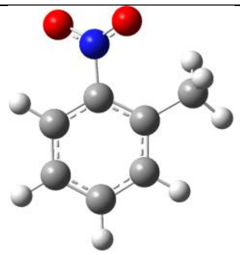

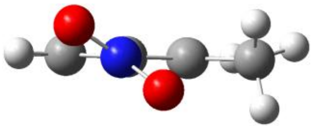

2-methylnitrobenzene CAS 88-72-2

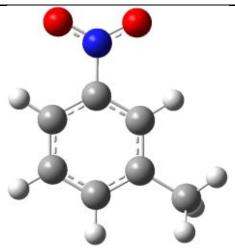

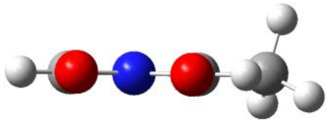

3-methylnitrobenzene CAS 99-08-1

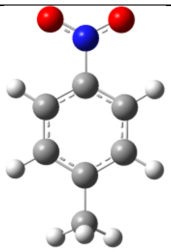

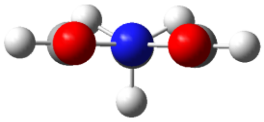

4-methylnitrobenzene CAS 99-99-0

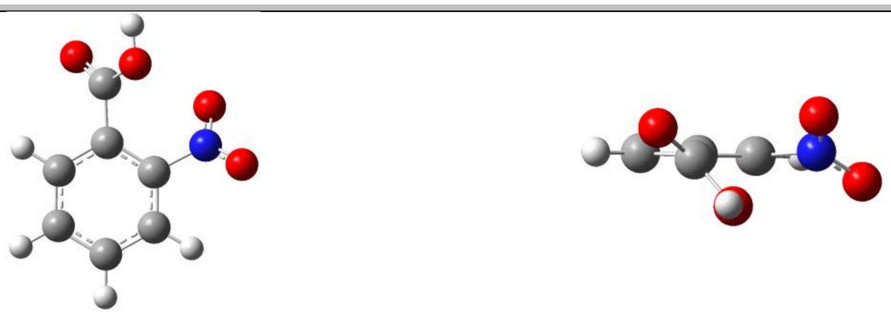

2-nitrobenzoic acid CAS 552-16-9

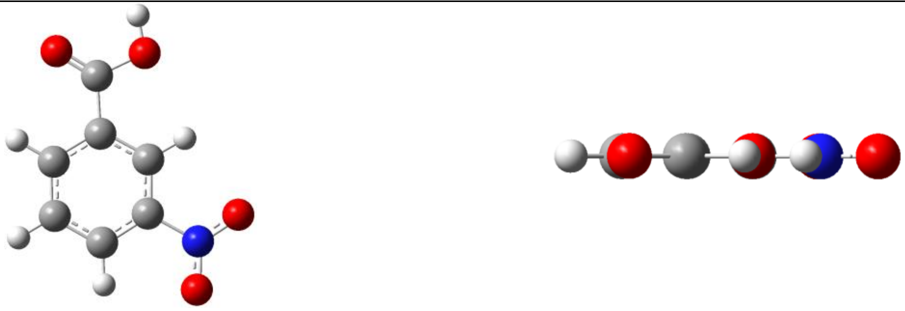

3-nitrobenzoic acid CAS 121-92-6

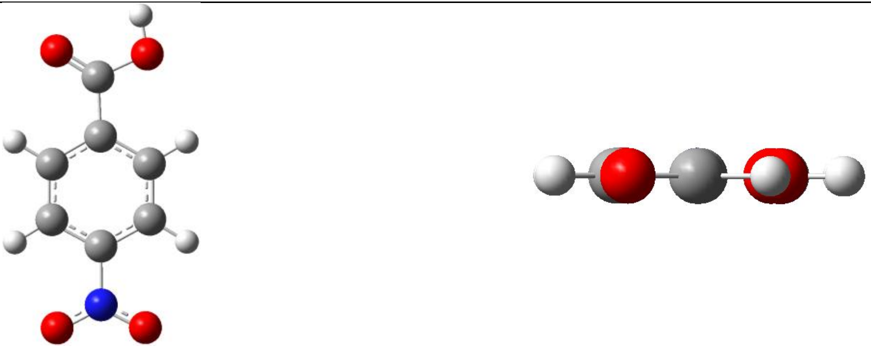

4-nitrobenzoic acid CAS 62-23-7

Table S11. Structures of the most stable conformers of methyl-nitrobenzoic acids

Front view	Top view
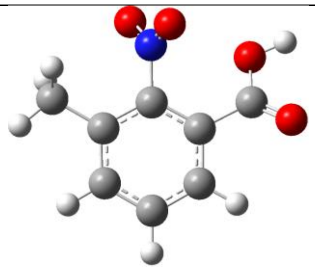<br>Relative energy, **0.0** kJ·mol^{-1}	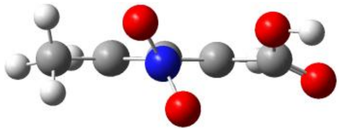
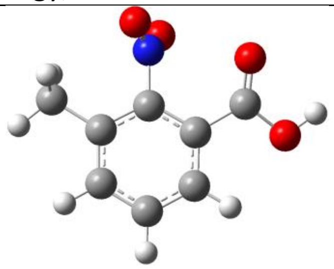<br>Relative energy, **0.6** kJ·mol^{-1}	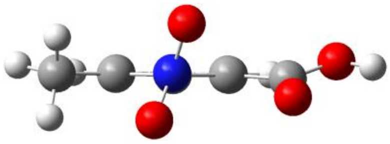
3-methyl-2-nitrobenzoic acid CAS 5437-38-7	
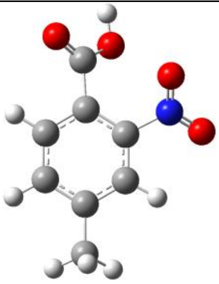	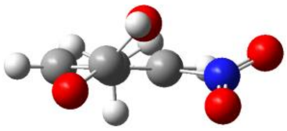
4-methyl-2-nitrobenzoic acid CAS 27329-27-7	
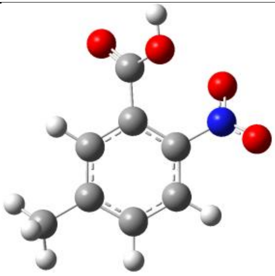	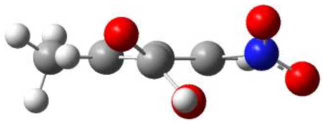
5-methyl-2-nitrobenzoic acid CAS 3113-72-2	
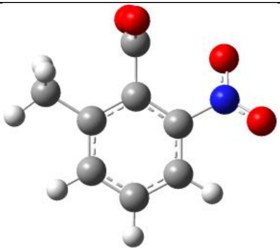	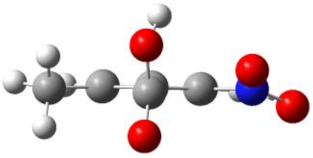
6-methyl-2-nitrobenzoic acid CAS 13506-76-8	

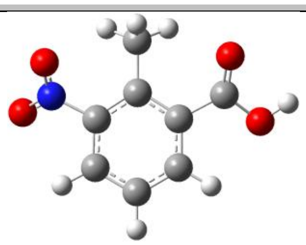
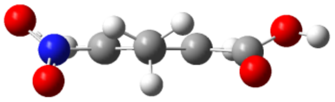

Relative energy, **0.0** kJ·mol^{-1}

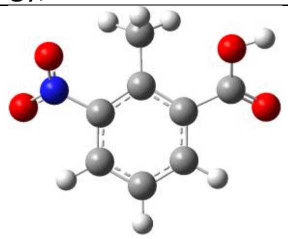
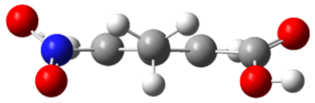

Relative energy, **3.5** kJ·mol^{-1}

2-methyl-3-nitrobenzoic acid CAS 1975-50-4

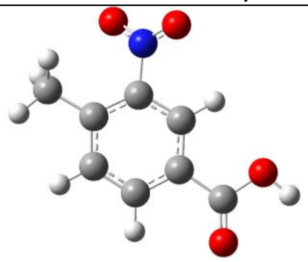
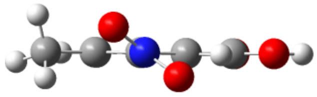

Relative energy, **0.0** kJ·mol^{-1}

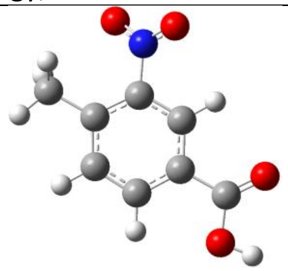
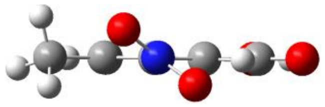

Relative energy, **0.9** kJ·mol^{-1}

4-methyl-3-nitrobenzoic acid CAS 96-98-0

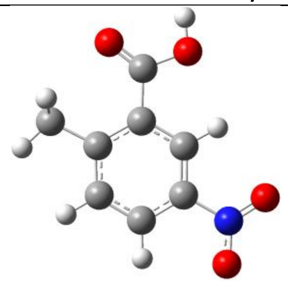
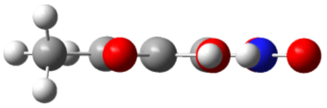

6-methyl-3-nitrobenzoic acid CAS 1975-52-6

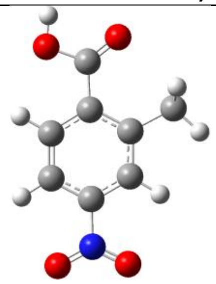
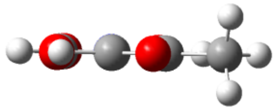

Relative energy, **0.0** kJ·mol^{-1}

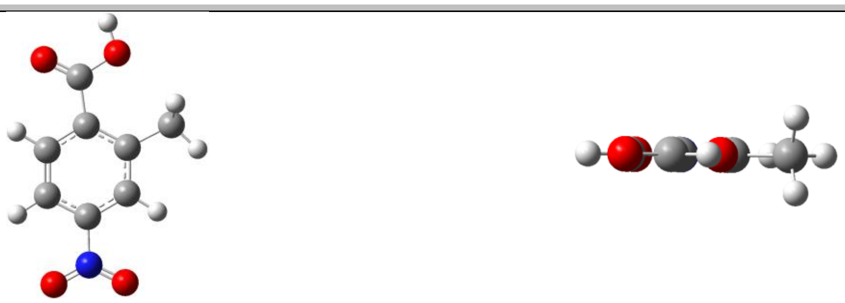

Relative energy, **4.9** kJ·mol^{-1}

2-methyl-4-nitrobenzoic acid CAS 1975-51-5

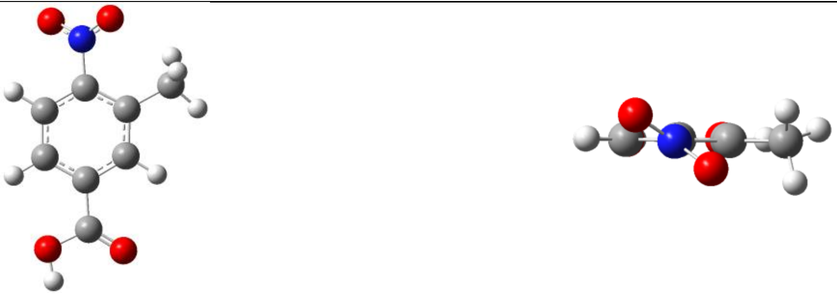

Relative energy, **0.0** kJ·mol^{-1}

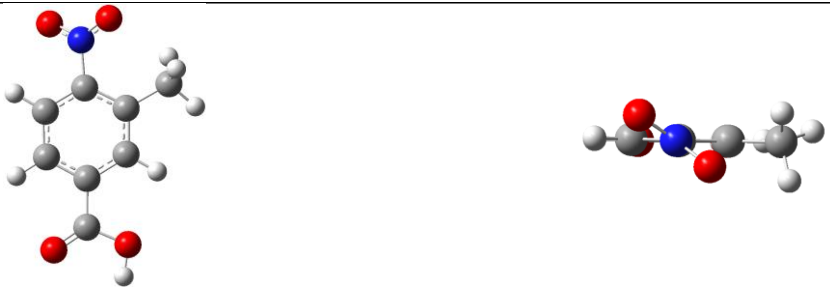

Relative energy, **0.1** kJ·mol^{-1}

3-methyl-4-nitrobenzoic acid CAS 3113-71-1

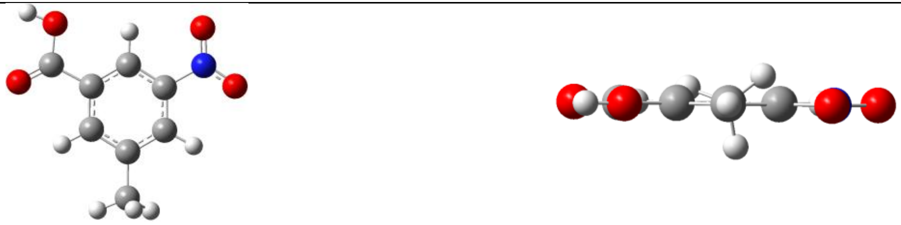

Relative energy, **0.0** kJ·mol^{-1}

3-methyl-5-nitrobenzoic acid CAS 113882-33-0

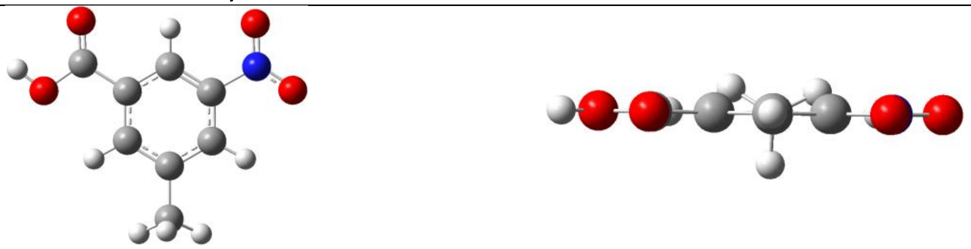

Relative energy, **1.3** kJ·mol^{-1}

3-methyl-5-nitrobenzoic acid CAS 113882-33-0

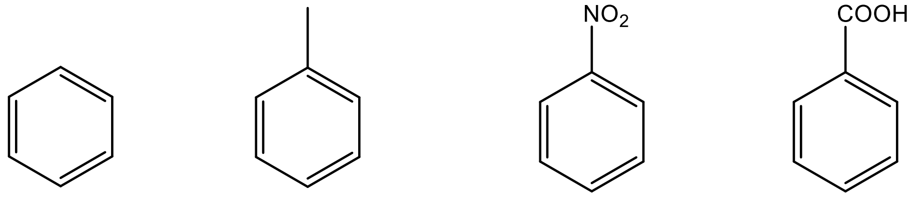

82.9 ± 0.9

(H → CH_3) = -32.8
50.1 ± 1.1
50.1 - 82.9 = -32.8

(H → NO_2) = -17.3
65.6 ± 1.6
65.6 - 82.9 = -17.3

-294.1 ± 2.2

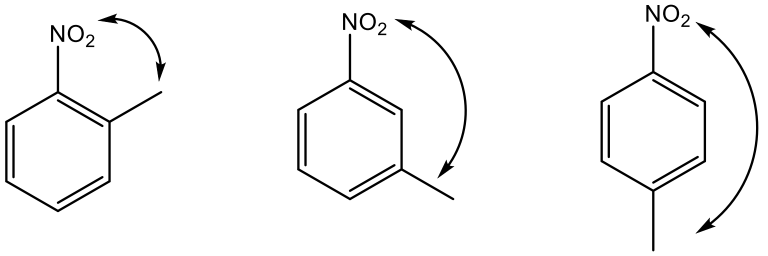

37.1 ± 1.0 (exp.)

Framework:
33.3 + (-17.3) + (-32.8) = 32.8
ortho (CH_3-NO_2)
37.1 - 32.8 = 4.3

29.0 ± 1.5 (exp.)

Framework:
33.3 + (-17.3) + (- 32.8) = 32.8
meta (CH_3-NO_2)
29.0 - 32.8 = -3.8

29.9 ± 1.1 (exp.)

Framework:
33.3 + (-17.3) + (- 32.8) = 32.8
para (CH_3-NO_2)
29.9 - 32.8 = -2.9

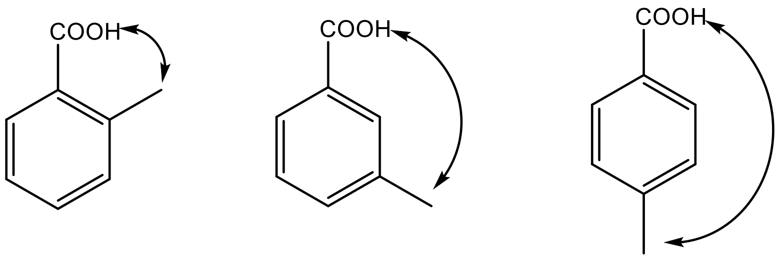

-320.3 ± 0.9 (exp.)

Framework:
-294.1 + (-32.8) = -326.9
ortho (CH_3-COOH)
-320.3 - (-326.9) = 6.6

-328.9 ± 0.9 (exp.)

Framework:
-294.1 + (-32.8) = -326.9
meta (CH_3-COOH)
-328.9 - (-326.9) = -2.0

-328.5 ± 2.6 (exp.)

Framework:
-294.1 + (-32.8) = -326.9
para (CH_3-COOH)
-328.5 - (-326.9) = -1.6

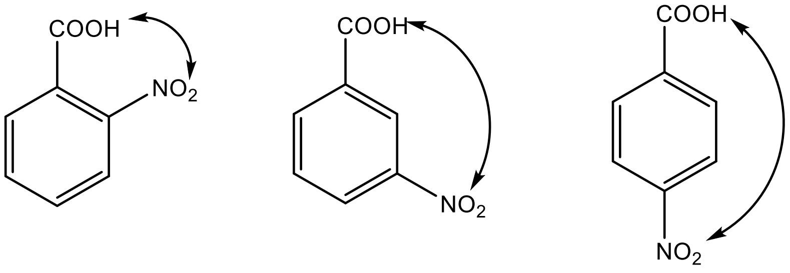

-280.3 ± 0.8 (exp.)

Framework:
-294.1 + (-17.3) = -311.4
ortho (NO_2-COOH)
-280.3 - (-311.4) = 31.1

-304.0 ± 0.6 (exp.)

Framework:
-294.1 + (-17.3) = -311.4
meta (NO_2-COOH)
-304.0 - (-311.4) = 7.4

-307.5 ± 1.0 (exp.)

Framework:
-294.1 + (-17.3) = -311.4
para (CH_3-COOH)
-307.5 - (-311.4) = 3.9

Fig. S3 Development of the increments and pairwise interactions of substituents on the benzene ring required for estimation of the gas-phase standard molar enthalpies of formation, of $\Delta_f H^o_m$(g, 298.15 K), of MNBAs using benzoic acid as the "centerpiece". All data are from Table S5 (in kJ·mol^{-1})

Table S12. Provenance and purity of the materials

Compound, CAS	Origin<br>Purity by supplier	DSC purity<br>(mass fraction)[a]
2-Methyl-3-nitrobenzoic acid, 1975-50-4	Alfa, 99%	0.999
2-Methyl-4-nitrobenzoic acid, 1975-51-5	Acros, 98%	0.993
3-Methyl-4-nitrobenzoic acid, 3113-71-1	Acros, 99%	0.995
5-Methyl-2-nitrobenzoic acid, 3113-72-2	Alfa, 98+%	0.993
6-Methyl-2-nitrobenzoic acid, 13506-76-8	Acros, 99%	0.995
4-Methyl-3-nitrobenzoic acid, 96-98-0	Acros, 99%	0.997
6-Methyl-3-nitrobenzoic acid, 1975-52-6	Acros, 99+%	0.998
3-Methyl-2-nitrobenzoic acid, 5437-38-7	Alfa, 98%	0.991
4-Methyl-2-nitrobenzoic acid, 27329-27-7	Sigma, 97%	0.989

[a] The final purity after purification, determined by DSC.

Samples for combustion calorimetry, Knudsen and transpiration experiments were purified using fractional sublimation under reduced pressure. The final purities of the samples were determined by DSC. Before starting the vapour pressure measurements, the samples were preconditioned in the transpiration saturator or in the Knudsen cell at 373 K in order to remove possible traces of volatile impurities. The small, residual amount of impurities found in the samples for the thermochemical studies belong to the isomeric methyl-nitrobenzoic acids with very similar thermodynamic properties, so that the traces of these isomers cannot in principle have any effect on the results for the main component.

ESI References

[34] B. Brunetti, A. Ciccioli, G. Gigli, A. Lapi, N. Misceo, L. Tanzi, S. Vecchio Ciprioti, "Vaporization of the prototypical ionic liquid BMImNTf(2) under equilibrium conditions: a multitechnique study", *Phys. Chem. Chem. Phys.* **2014**, *16*, 15653-15661.

[35] M. Knudsen, *Ann. Phys.* **1909**, *29*, 179.

[36] M. A. V. Ribeiro da Silva, M. J. S. Monte, L. M. N. B. F. Santos, "The design, construction, and testing of a new Knudsen effusion apparatus", *J. Chem. Thermodyn.* **2006**, *38*, 778-787.

[37] D. B. Newell, Cabiati, F., Fischer, J., Fujii, K., Karshenboim, S.G., Margolis, H.S, de Mirandes, E., Mohr, P.J., Nez, F., Pachucki, K., Quinn, T.J., Taylor, B.N., Wang, M., Wood, B.M., Zhang, Z., "The CODATA 2017 values of h, e, k, and NA for the revision of the SI", *Metrologia* **2018**, *55*.

[38] J. S. Chickos, D. G. Hesse, J. F. Liebman, "A Group Additivity Approach for the Estimation of Heat Capacities of Organic Liquids and Solids at 298 K", *Struct. Chem.* **1993**, *4*, 261-269.

[39] D. Kulikov, S. P. Verevkin, A. Heintz, "Determination of Vapor Pressures and Vaporization Enthalpies of the Aliphatic Branched C5 and C6 Alcohols", *J. Chem. Eng. Data* **2001**, *46*, 1593-1600.

[40] S. P. Verevkin, V. N. Emel'yanenko, "Transpiration method: Vapor pressures and enthalpies of vaporization of some low-boiling esters", *Fluid Phase Equilib.* **2008**, *266*, 64-75.

[41] V. N. Emel'yanenko, D. H. Zaitsau, E. Shoifet, F. Meurer, S. P. Verevkin, C. Schick, C. Held, "Benchmark Thermochemistry for Biologically Relevant Adenine and Cytosine. A Combined Experimental and Theoretical Study", *J. Phys. Chem. A* **2015**, *119*, 9680-9691.

[42] H. A. Jones, I. L. r, G. M. J. Mackay, "The Rates of Evaporation and the Vapor Pressures of Tungsten, Molybdenum, Platinum, Nickel, Iron, Copper and Silver", *Phys. Rev.* **1927**, *30*, 201-214.

[43] M. V. Roux, M. Temprado, J. S. Chickos, Y. Nagano, "Critically Evaluated Thermochemical Properties of Polycyclic Aromatic Hydrocarbons", *J. Phys. Chem. Ref. Data* **2008**, *37*, 1855-1996.

[44] S. P. Verevkin, V. N. Emel'yanenko, V. Diky, O. V. Dorofeeva, "Enthalpies of formation of nitromethane and nitrobenzene: New experiments vs. quantum chemical calculations", *J. Chem. Thermodyn.* **2014**, *73*, 163-170.

[45] J. Pedley, *Thermochemical data and structures of organic compounds, Vol. 1*, CRC Press, **1994**.

[46] S. P. Verevkin, C. Schick, "Substituent Effects on the Benzene Ring. Determination of the Intramolecular Interactions of Substituents in tert-Alkyl-Substituted Catechols from Thermochemical Measurements", *J. Chem. Eng. Data* **2000**, *45*, 946-952.

[47] V. N. Emel'yanenko, S. P. Verevkin, A. Heintz, "The Gaseous Enthalpy of Formation of the Ionic Liquid 1-Butyl-3-methylimidazolium Dicyanamide from Combustion Calorimetry, Vapor Pressure Measurements, and Ab Initio Calculations", *J. Am. Chem. Soc.* **2007**, *129*, 3930-3937.

[48] W. N. Hubbard, D. W. Scott, G. Waddington, in *Experimental Thermochemistry, Vol. 1* (Ed.: F. D. Rossini), Interscience, New York, **1956**, pp. 75-127.

[49] S. Sunner, M. Månsson, *Combustion Calorimetry: Experimental Chemical Thermodynamics*, Pergamon, **2016**.

[50] K. Fang, G. Li, Y. She, "Metal-Free Aerobic Oxidation of Nitro-Substituted Alkylarenes to Carboxylic Acids or Benzyl Alcohols Promoted by NaOH", *J. Org. Chem.* **2018**, *83*, 8092-8103.

[51] A. B. Singaraju, K. Nguyen, D. C. Swenson, M. Iyer, R. V. Haware, L. L. Stevens, "Reorganized, weak C–H···O interactions directly modify the mechanical properties and compaction performance of a series of nitrobenzoic acids", *Cryst.Eng.Comm.* **2017**, *19*, 2526-2535.

[52] S. P. Verevkin, "Thermochemistry of phenols: quantification of theortho-,para-, andmeta-interactions in tert-alkyl substituted phenols", *J. Chem. Thermodyn.* **1999**, *31*, 559-585.

[53] S. H. Mir, Y. Takasaki, E. R. Engel, S. Takamizawa, "Enhancement of dissipated energy by large bending of an organic single crystal undergoing twinning deformation", *RSC Advances* **2018**, *8*, 21933-21936.

[54] D. H. Andrews, G. Lynn, J. Johnston, "The heat capacities and heat of crystallization of some isomeric aromatic compounds", *J. Am. Chem. Soc.* **1926**, *48*, 1274-1287.
